# Comprehensive genomic survey, structural classification and expression analysis of C2H2 zinc finger protein gene family in *Brassica rapa* L.

**DOI:** 10.1371/journal.pone.0216071

**Published:** 2019-05-06

**Authors:** Intikhab Alam, Khadija Batool, Dong-Li Cui, Yan-Qing Yang, Yun-Hai Lu

**Affiliations:** 1 Key Laboratory of Ministry of Education for Genetics, Breeding and Multiple Utilization of Crops, College of Crop Science, Fujian Agriculture and Forestry University, Fuzhou, China; 2 State Key Laboratory of Ecological Pest Control for Fujian and Taiwan Crops, College of Life Sciences, Key Lab of Biopesticides and Chemical Biology, Fujian Agriculture and Forestry University, Fuzhou, China; Huazhong University of Science and Technology, CHINA

## Abstract

C2H2 zinc finger protein (ZFP) genes have been extensively studied in many organisms and can function as transcription factors and be involved in many biological processes including plant growth and development and stress responses. In the current study, a comprehensive genomics analysis of the C2H2-ZFP genes in *B*. *rapa* was performed. A total of 301 *B*. *rapa* putative C2H2-ZFP (BrC2H2-ZFP) genes were identified from the available *Brassica* genome databases, and further characterized through analysis of conserved amino acid residues in C2H2-ZF domains and their organization, subcellular localization, phylogeny, additional domain, chromosomal location, synteny relationship, Ka/Ks ratio, and expression pattern. We also analyzed the expression patterns of eight *B*. *rapa* C2H2-ZFP genes under salt and drought stress conditions by using qRT-PCR technique. Our results showed that about one-third of these *B*. *rapa* C2H2-ZFP genes were originated from segmental duplication caused by the WGT around 13 to 17 MYA, one-third of them were highly and consecutively expressed in all tested tissues, and 92% of them were located in nucleus by prediction supporting then their functional roles as transcription factors, of which some may play important roles in plant growth and development. The Ka/Ks ratios of 264 orthologous C2H2-ZFP gene pairs between *A*. *thaliana* and *B*. *rapa* were all, except two, inferior to 1 (varied from 0.0116 to 1.4919, with an average value of 0.3082), implying that these genes had mainly experienced purifying selection during species evolution. The estimated divergence times of the same set of gene pairs ranged from 6.23 to 38.60 MY, with an average value of 18.29 MY, indicating that these gene members have undergone different selective pressures resulting in different evolutionary rates during species evolution. In addition, a few of these *B*. *rapa* C2H2-ZFPs were shown to be involved in stress responses in a similar way as their orthologs in *A*. *thaliana*. Comparison between *A*. *thaliana* and *B*. *rapa* orthologous C2H2-ZFP genes showed that the majority of these C2H2-ZFP gene members encodes proteins with conserved subcellular localization and functional domains between the two species but differed in their expression patterns in five tissues or organs. Thus, our study provides valuable information for further functional determination of each C2H2-ZFP gene across the *Brassica* species, and may help to select the appropriate gene targets for further in-depth studies, and genetic engineering and improvement of *Brassica* crops.

## Introduction

The zinc finger proteins (ZFPs) are one of the most common groups of proteins widely existing in all eukaryotic organisms. Zinc finger proteins were known to be involved in many cellular processes including DNA-binding, RNA-binding, protein-protein interaction, and protein folding, *etc*. [1–3], and play important roles in plant growth and development as well as response to various environmental stresses [4–7]. The zinc finger (ZF) structure is maintained by the zinc ion, which coordinates conserved cysteine and/or histidine residues in different combinations. According to the type and order of these zinc coordinated residues, the ZFs can be classified into different types, such as, C2H2, C2C2, and C3H1 domains coordinating one zinc ion, whereas C3HC4 RING finger, PHD and LIM domains coordinating two zinc ions [8–10]. As high as 30 types of ZFs were approved in human genome [9].

The C2H2-ZFs, also called classical ZFs, were the first class to be characterized among all the types of ZFs [9]. The C2H2-ZF domain was defined as about 30 amino acids with two conserved Cys and two His residues which bound to one Zn^2+^ atom tetrahedrally and form a structure as C-X_2-4_–C-X_3_–F-X_5_-L-X_2_–H-X_3-5_–H, where X represents any amino acid [2,11]. Plant C2H2 zinc finger proteins differ from other eukaryotic organisms by a long and diverse space between two zinc fingers, while in animal, the spaces between two tandem repeated zinc fingers are often short (seven amino acid residues) and known as the HC links [8], as it was found in Drosophila and yeast [12]. Additionally, the α-helix in the plant zinc finger domain has a very conserved sequence, QALGGH, which is absent in other organisms, and probably the result of evolution through natural selection for a specific regulatory process unique in plants [7,13]. C2H2-ZFP family has been reported in several plant genomes, including 179 members in *Arabidopsis thaliana* [14], 189 in rice [15], 321 in soybean [16], 211 in maize [17], 124 in foxtail millet [18], 122 in durum wheat [19], 118 in tobacco [20], and 109 in poplar [21].

Recently, extensive studies showed that most of C2H2-ZFPs functioned as transcription factors and were involved in various biological processes, including plant growth and development, hormone signaling and stress responses in many plants species [4,18,22,23]. For example, in rice ZFP179 is involved in salt tolerance [24], and ZFP245 can increase the tolerance to cold, drought and oxidative stresses [25]. In soybean, SCOF-1 is involved in low-temperature tolerance [26]. In *Brassica napus*, BnLATE is involved in silique shattering [27]. In *A*. *thaliana*, RHL41 is involved in light stress [24]. In *Capsicum annuum*, CAZFP1 is involved in pathogen defense [28]. ZFP252 can enhance drought and salt tolerance in rice [29]. SIZF2 can influence flower and leaf shape in *A*. *thaliana*, and enhance salt tolerance in tomato [30]. BcZAT12 can improve tolerance to drought in tomato [31]. ZFP1 positively regulates tolerance to both cold and drought stress in *A*. *thaliana* [32], GsZFP1 is involved in ABA signaling, by reduced ABA sensitivity and decreased stomata size in transgenic *A*. *thaliana* plants [33]. GmWRKY49 is a positive regulator of salinity tolerance in soybean [34]. SIZF3 can significantly increase the level of AsA and enhance salt stress tolerance [35], GhSTOP1 can accelerate root growth and is essential for aluminum and proton stress tolerance in *A*. *thaliana* [36]. SGR5 is involved in primary events of geotropism in inflorescence stems in *A*. *thaliana* [37]. SUPERMAN (SUP), controls the boundary between the stamen and carpel whorls [38–40], prevents class B gene expression and promotes stem cell termination in the fourth whorl of *A*. *thaliana* flowers [41]. TRANSPARENT TESTA1 (TT1) controls seed endothelium differentiation in *A*. *thaliana* [42].

*Brassica* genus includes numerous economically important species with notable morphological diversity due to long term evolution [43], providing human nutrition in the form of vegetables, oil, condiments, dietary fiber, and vitamin C [44]. Among the *Brassica* species, *Brassica rapa* (n = 10, AA genome, 529 Mb genome size) is one of the most economically important diploid progenitor species which contributes the ‘A’ genome to the allopolyploid oilseed crops, *B*. *napus* (n = 19, AACC) and *B*. *juncea* (n = 18, AABB) [45]. *B*. *rapa* also serves as a model plant for studies related to genomic or evolution due to small genome size (about 529 Mbp) [46] and exhibits very close relation with the model plant *A*. *thaliana*. Additionally, it experienced a whole-genome triplication (WGT) event since its divergence from *A*. *thaliana* about 13 to 17 million years ago (MYA) [47]. The availability of whole *B*. *rapa* genome sequences offers an unprecedented opportunity for genome-wide identification, evolution and functional analysis of various important gene families in this species.

In this study, we performed the genome-wide identification of the C2H2-ZFP gene family in *B*. *rapa*. Comprehensive analyses were carried out based on phylogeny, chromosome location, and syntenic relationship between *B*. *rapa* and *A*. *thaliana*. Furthermore, expression analysis was carried out based on RNA-seq data of this gene family in different tissues, and the expression patterns of a few members under salt and drought stress treatments were also investigated through qRT-PCR. Our results provide a solid foundation for further functional characterization of C2H2-ZFP genes and provide helpful information in the field of genetics and the evolution of this model species.

## Materials and methods

### Identification and classification of C2H2-ZFPs in *B*. *rapa*

We identified the C2H2-ZFPs in *B*. *rapa* using two different approaches. First, all 176 known C2H2-ZFPs in *A*. *thaliana* [14] were retrieved from the TAIR database (http://www.arabidopsis.org/) and used as queries to BLASTp against the whole *B*. *rapa* genome annotation data deposited at the *Brassica* Database (BRAD, ver. 1.5, http://brassicadb.org/brad/). Second, each type of representative *A*. *thaliana* C2H2-ZF domains was used as queries to BLASTp against the same database in order to fully identify the C2H2-ZFPs. In both cases, the retrieved irredundant sequences were submitted to SMART database (http://smart.embl-heidelberg.de/) with the chosen option of Pfam domains to confirm the presence of C2H2 domains, combined by manual inspection of each C2H2 zinc finger domain sequence [48]. We determined the C2H2 type for each identified *B*. *rapa* C2H2 domain, according to the plant-specific amino acid residues and distances between two∼nine C2H2-ZF domains, as have been previously adopted in soybean [16]. C2H2-ZFPs containing two∼nine C2H2 domains, and every two nearby domains were linked by less than 12 amino acid residues, were classified as Tandem ZFs with two subsets assigned as Br-t1-SF and Br-t2-SF, respectively; while C2H2-ZFPs containing a single and/or two∼four C2H2 domains and every two nearby domains alienated by more than 11 amino acid residues were classified as dispersed C2H2-ZFPs. Furthermore, different types of C2H2-ZFs domains were classified based on the variation of the plant-specific conserved amino acid sequence “QALGGH” and distances between metal ligands [2,21,49]. For example, C2H2-ZF Q-type domains were defined as X2-C-X2-C-X7-QALGGH-X3-H. M-type (M1∼M5) domains were defined for those with one∼five degraded amino acids in the plant-specific conserved amino acid sequence “QALGGH” and certain modifications in the spacing between the two cysteine’s and two histidines. The Z-type domains were defined as those with more than 12 (Z1) and less than 12 (Z2) in their spacing between the second cysteine and the first histidine. The D-type domains were defined as those missing the second histidine in the C2H2-ZF domain compared with the other three types. According to these defined C2H2-ZF types, C2H2-ZFPs containing a single ZF domain were further classified into four clearly distinguishable subsets (Br-1i-Q-SF, Br-1i-M-SF, Br-1i-Z-SF, and Br-1i-D-SF). C2H2-ZFPs containing two Q-types, two M-types or two Z-types of C2H2-ZF domains were defined as Br-2i-Q-SF, Br-2i-M-SF or Br-2i-Z-SF, respectively, while the C2H2-ZFPs containing two different types of C2H2-ZF domains were classified as Br-2i-Mix-SF. All C2H2-ZFPs containing three or four dispersed ZFs were classified into the subsets of Br-3i-SF or Br-4i-SF, respectively. For each identified *B*. *rapa* C2H2-ZFP, their size, amino acid properties, charge, molecular weight (kDa) and isoelectric points (pI) were determined using the online available ProtParam tool (http://web.expasy.org/protparam/) [50], and their subcellular localization was predicted by using the online cello2go software (http://cello.life.nctu.edu.tw/cello2go/) in combination with the WoLF PSORT program (https://www.genscript.com/wolf-psort.html).

### Multiple sequence alignments, and phylogenetic analysis

The C2H2-ZF domains retrieved from proteins were aligned by using program Clustal W and manually edited by BioEdit software to specify metal ligand positions. Based on *B*. *rapa* C2H2-ZFP sequences, phylogenetic trees were generated by using the MEGA 7.0 software with the Maximum Likelihood (ML) method and a bootstrap analysis of 1000 replicates.

### Chromosome location of C2H2-ZFPs protein genes in *B*. *rapa*

The chromosome location data of each *B*. *rapa* C2H2-ZFP genes were downloaded from the BRAD database. Those genes that were assigned to unassembled genomic scaffolds (absence of chromosomal position) were removed from the dataset while the remaining genes were mapped into the specific chromosomes of *B*. *rapa* by using Map chart 2.3v software. Tandem repeated and segmentally duplicated genes were indicated by different color lines.

### Syntenic relationships between *B*. *rapa* and *A*. *thaliana* C2H2 zinc finger protein genes

For each predicted *A*. *thaliana* and *B*. *rapa* C2H2-ZFP, we used the Search Syntenic Gene function of the BRAD database to find out its *A*. *thaliana* ortholog (if existed). On the other hand, for each known *A*. *thaliana* C2H2-ZFP gene, we used the same function to find out its *B*. *rapa* ortholog(s) (if existed). In each query, the information about the corresponding orthologous gene name(s) in *A*. *thaliana* and *B*. *rapa*, their localization on tPCK (Translocation Proto-Calepineae Karyotype) chromosomes and ancestral chromosome blocks, LF (least fractioned), MF1 (medium fractionated) and MF2 (most fractionated) subgenomes [51–53], as well as the eventual tandem repeats in the two species were recorded. The C2H2-ZFP full-length amino acid sequences were aligned by using program Clustal Omega (http://www.ebi.ac.uk/Tools/msa/clustalo/) and then the synonymous rate (Ks), non-synonymous rate (Ka), and evolutionary constriction (Ka/Ks) were calculated by using the PAL2NAL by using the codeml program in PAML (http://www.bork.embl.de/pal2nal/index.cgi?example=Yes#RunP2N) [54,55]. The divergence time was calculated through formula T = Ks/2R, where T mentions to divergence time, Ks mentions to the synonymous substitutions per site, R is intended for the divergence rate of nuclear genes from plants, R-value is considered as 1.5 × 10^−8^ synonymous substitutions per site per year in case of dicotyledonous plants [56].

### Expression pattern of C2H2-ZFP genes in *B*. *rapa*

The RNA-seq data of gene expression of six tissues (callus, root, stem, leaf, flower, and silique) of the *B*. *rapa* accession Chiifu-401–42 was retrieved from the Gene Expression Omnibus (GEO) database of NCBI (http://www.ncbi.nlm.nih.gov/geo/) using the accession number GSE43245 [57]. The expression values (Fragments Per Kilobase of exon model per Million mapped, FPKM) of identified *B*. *rapa* C2H2-ZFP genes were extracted from the dataset and submitted to clustering analysis using Cluster software v3.0 (http://bonsai.hgc.jp/mdehoon/software/cluster/) with log2-transformed FPKM values, Euclidean distances, and the average linkage clustering method. The clustering tree together with the gene expression heat map was generated by using the Java Tree view software (Version1.1.5r2, http://jtreeview.sourceforge.net/).

### Preparation of plant material and qRT-PCR analyses

For plant samples preparation, we cultivated *B*. *rapa* accession Chiifu-401–42 seeds were sterilized and sown in a Petri dish with moisture-absorbent filter papers and incubated at 25°C. The germinated seeding was then transferred into plastic pots containing growth medium with vermiculite and peat 3:1 grown in a greenhouse at 22 °C with a photoperiod of 16/8 h for light/dark. 21-days-old seedlings were used for different abiotic treatments. For salinity and PEG treatments, the plants were irrigated with 200 mM NaCl and 20% (w/v) polyethylene glycol (PEG 6000). Then, leaves (third, fourth leaves) from control and stressed plants were harvested after 0, 1, 3, and 24 h of treatments and immediately immersed in liquid nitrogen, and stored at -80 °C until RNA extraction use. For each treatment, three biological replicates were prepared to decrease the error rate.

Total RNA was isolated from the frozen leaves of each sample of *B*. *rapa* using a Plant RNA extraction Kit, according to the manufacturer’s instructions (OMEGA, China). The quality of RNA was checked by agarose gel electrophoresis, and also by using the NanoDrop 2000 Spectrophotometer (Thermo Fisher Scientific, Inc., Waltham, MA, USA). First-strand cDNA was synthesized using 1μg of total RNA per sample with the cDNA Synthesis Kit from TaKaRa Bio Inc. (Dalian, China). The reverse transcription products were diluted 20-fold and stored at −20 °C prior to analysis. Gene-specific primers for the selected *B*. *rapa* C2H2-ZFP genes were designed using Primer3Plus software (http://www.primer3plus.com/). The *B*. *rapa* Actin-2 gene (GenBank accession number XM_018658258) was used as an internal reference gene. The primers used for qRT-PCR and their expected amplification product size are summarized in S6 Table. The qRT-PCR analysis was performed on an ABI 7500 Fast Real-time PCR amplification system (Applied Biosystems, Foster, CA, USA). The analysis was carried out in a total volume of 20 μL containing 2 μL template of cDNA, 0.8 μL of the forward and reverse primers (10 μM), 10μL of SYBR Green PCR Master (ROX) (Roche, Shanghai, China), 6.4 μL of sterile distilled water. The PCR amplification parameters were as following: 95 °C for 1 minute, followed by 40 cycles of 95 °C for 15 s, and 60 °C for 70 s. For each sample, three replicates were run to compute the average Ct values. The data were analyzed using the 2−ΔΔCt methods [58]. Relative gene expression levels were normalized against the expression of the housekeeping gene BrActin-2. The significance of differences with P<0.05 among relative expression levels of the genes were statistically analyzed (Tukey HSD test) by using IBM SPSS version 22.

## Results

### Genome-wide identification and classification of C2H2-ZFPs in *B*. *rapa*

To identify the C2H2-ZFPs, we searched the *Brassica* genome database and further confirmed through Smart database and manual inspection. A total of 301 putative C2H2-ZFPs containing 534 C2H2 domains were identified in *B*. *rapa* genome, and summarized in S1 Table together with their relative information. Furthermore, the 534 C2H2-ZF domains were classified into four categories (named Q, M, Z and D) based on the variation of the plant-specific conserved amino acid sequence “QALGGH” and distances between metal ligands (Table 1, S2 Table). S1 Fig illustrated the multiple sequence alignments of all the identified 534 C2H2-ZFs in *B*. *rapa*.

**Table 1:** The types and sub-types of C2H2-ZF domains and their characteristics in *Brassica rapa*. X represents any amino acid and the number represents the consensus spacing between the conserved amino acid residues.

Type of ZF	Sub-type	Conserved motif sequence description	Conserved spacing
**Q**	-	QALGGH	X2-C-X2-C-X7-QALGGH-X3-H
M	M1	1 degraded amino acid in QALGGH	X2-C-X2-C-X12-H-X(3,4)-H
	M2	2 degraded amino acids in QALGGH	X2-C-X2-C-X12-H-X(3,4)-H
	M3	3 degraded amino acids in QALGGH	X2-C-X(2,4)-C-X12-H-X(3,4,5)-H
	M4	4 degraded amino acids in QALGGH	X2-C-X(1,2,4)-C-X12-H-X(1,3,4,5,7)-H
	M5	5 degraded amino acids in QALGGH	X2-C-X(2,4)-C-X12-H-X(3,4,5,8)-H
Z	Z1	-	X2-C-X(2,3,4)-C-X(>12)-H-X(2,3,4,5)-H
	Z2	-	X2-C-X2-C-X(<12)-H-X(3,4)-H
D	-	-	X2-C-X(2,4)-C-X(12,13)-H-X2

**Fig 1:**
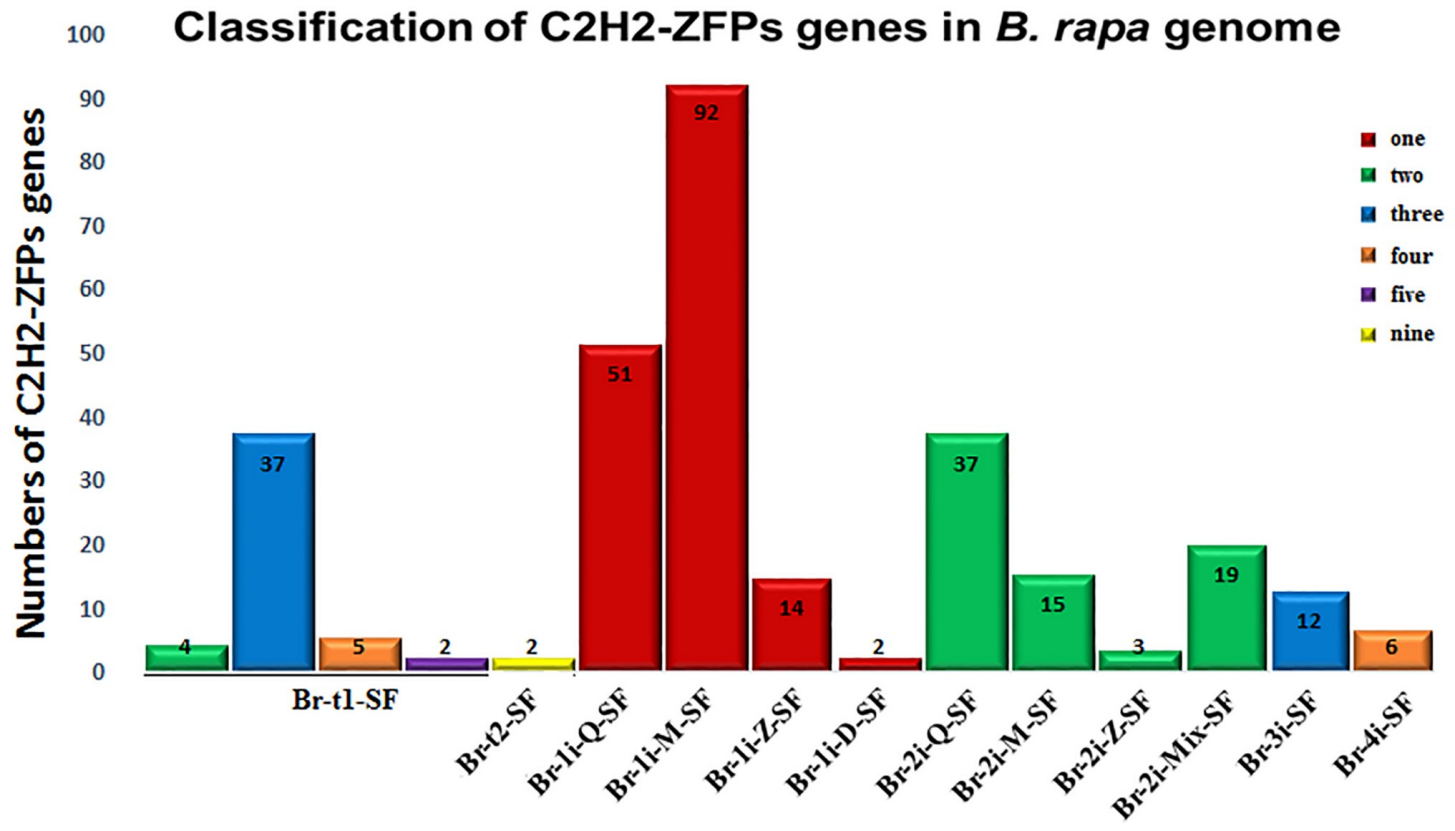
Classification of C2H2-ZFP genes in *Brassica rapa* genome. Numbers of C2H2-ZFPs in the 12 different subsets and containing 1–5 and 9 C2H2-ZF domains were shown by different colors. For the detailed information, see S2 Table.

Among the identified 301 putative C2H2-ZFPs, fifty (16.6%) were classified as tandem C2H2-ZFPs, including two subsets, i.e., Br-t1-SF (48 ZFPs) and Br-t2-SF (two ZFPs). Among the Br-t1-SF subset, 37 C2H2-ZFPs contained three C2H2 domains, 5 had four C2H2 domains, four had two C2H2, and two possessed five C2H2 domains while other subset Br-t2-SF (two ZFPs) possessed nine C2H2 domains (Fig 1). Furthermore, 251 (83.38%) ZFPs were identified as isolated ZFPs, which were further divided into ten subsets: Br-1i-Q-SF (51, 20.31%), Br-1i-M-SF (92, 36.65%), Br-1i-Z-SF (14, 5.57%), Br-1i-D-SF (2, 0.8%), Br-2i-Q-SF (37, 14.74%), Br-2i-M-SF (15, 5.98%), Br-2i-Z-SF (3, 1.19%), Br-2i-Mix-SF (19, 7.57%), Br-3i-SF (12, 4.78%), and Br-4i-SF (6, 2.39%) (Fig 1). Among the 251 isolated ZFPs, 159 contained a single C2H2 domain, 74 contained two, 12 contained three C2H2 domains, and six contained four C2H2 domains (Fig 1, S2 Table). Furthermore, 63 contained a single Q-type C2H2-ZF domain (51 in Br-1i-Q-SF, seven in Br-2i-Mix-SF, and five in Br-3i-SF), followed by 46 containing two Q-type C2H2-ZF domains (37 in Br-2i-Q-SF, six in Br-3i-SF and three in Br-4i-SF, respectively) and one containing four Q-type C2H2-ZF domains (Br-4i-SF) (S2 Table).

Analysis of the 301 *B*. *rapa* C2H2-ZFP sequences showed that their length of amino acid ranged from 81 to 1673 aa, with computed molecular weight ranged from 9.34 kDa to 190.32 kDa, and isoelectric points (pIs) ranged from 4.42 to 10.46. The predictions of subcellular localization showed that 277 (92%) members of *B*. *rapa* C2H2-ZFPs were localized in nucleus, 11 (3.65%) C2H2-ZFs were localized in cytoplasm, and 13 (4.31%) were located in different organelles (S1 Table).

### Phylogenetic analysis of the classified *B*. *rapa* C2H2-ZFP genes

**Fig 2:**
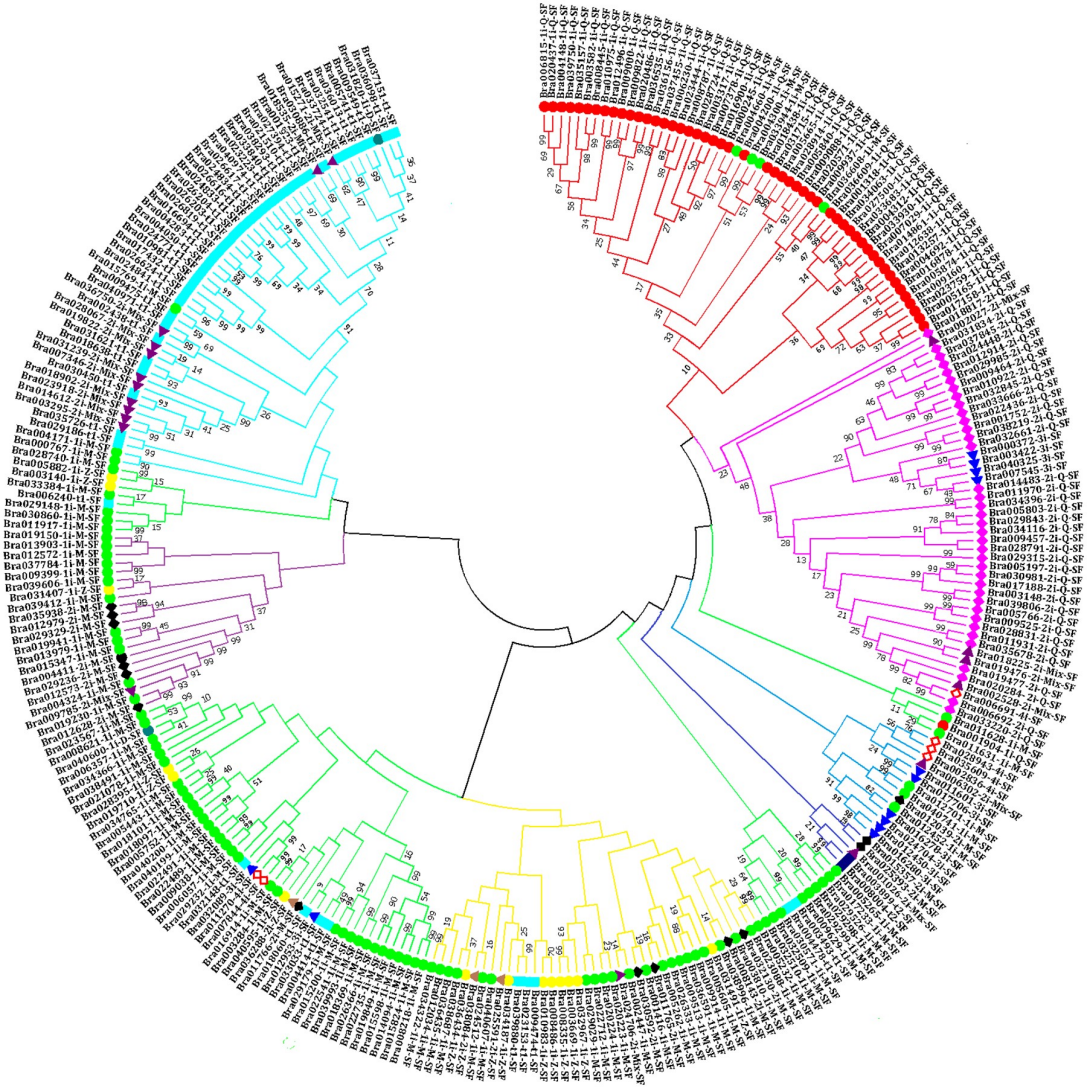
Phylogenetic tree analysis of the classified *Brassica rapa* C2H2-ZFPs. The tree was determined by using MEGA7.0 software with the Neighbor–Joining (NJ) algorithm and a bootstrap analysis of 1,000 replicates. The 12 classified *B*. *rapa* C2H2-ZFP subsets were clustered and indicated by different colors, respectively.

In order to investigate the phylogenetic relationships of 301 C2H2-ZFPs in *B*. *rapa*, a phylogenetic tree was constructed based on the multiple sequence alignment of all identified C2H2-ZFPs by using the ClustalW program implemented in MEGA 7.0 software (Fig 2). The phylogenetic relationships of 12 main C2H2-ZFP types (Br-t1-SF, Br-t2-SF, Br-1i-Q-SF, Br-1i-M-SF, Br-1i-Z-SF, Br-1i-D-SF, Br-2i-Q-SF, Br-2i-M-SF, Br-2i-Z-SF, Br-2i-Mix-SF, Br-3i-SF and Br-4i-SF) were clearly illustrated by the tree in Fig 2. The C2H2-ZFPs that had been classified into a same subset according to their contained C2H2-ZFs (Fig 1) tended to be also clustered together in the phylogenetic tree, although most of the main branches have often low bootstrap values (<50%) (Fig 2). Interestingly, a few exceptions were also observed as shown in Fig 2 where the differently colored points were occasionally found among a given C2H2-ZFP type.

### Chromosomal distribution and gene duplication

**Fig 3:**
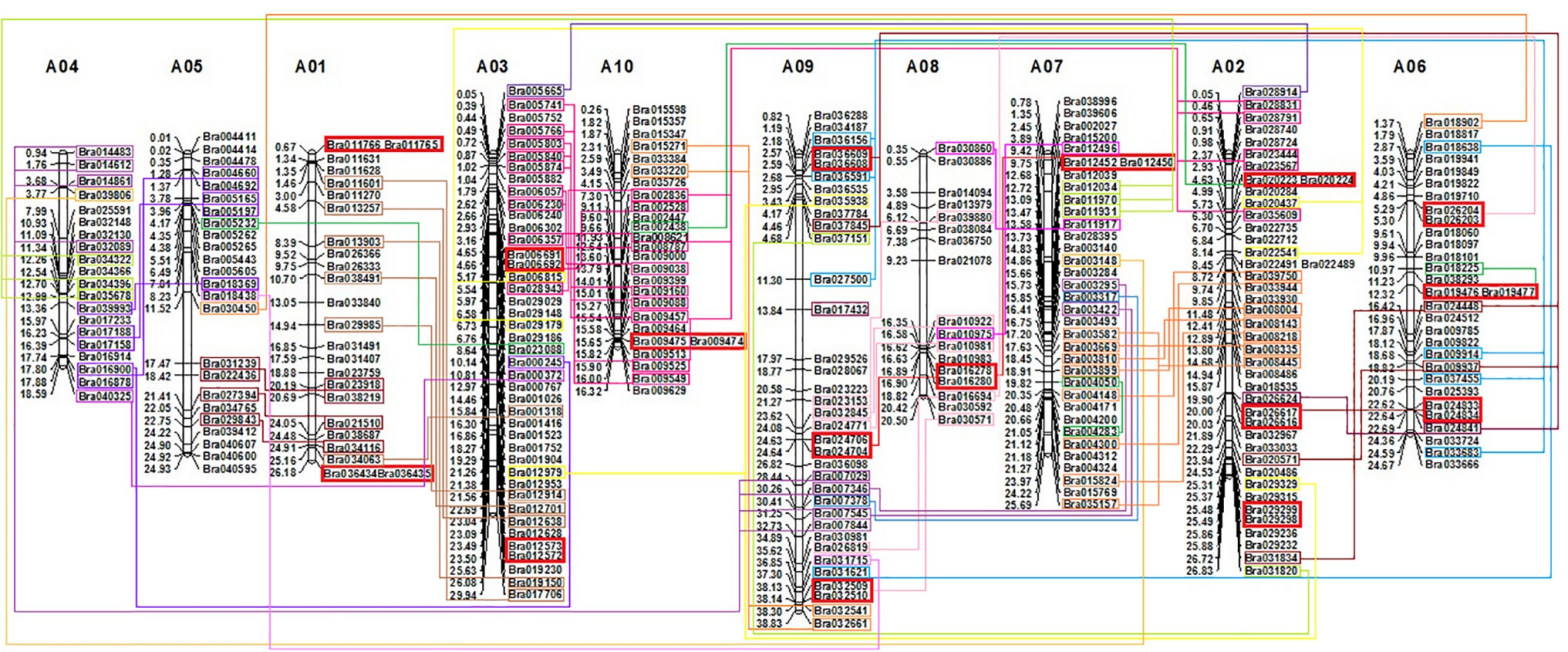
Distribution of 295 C2H2-ZFP genes on 10 chromosomes of *Brassica rapa*. The 295 C2H2-ZFP genes unevenly located on each conserved collinear blocks of the chromosomes. Chromosome number (A01-A10) is indicated at the *top* of each chromosome. Gene name is indicated on the *right side* of each chromosome. The physical position (Mb) of each mapped gene is indicated on the *left side* of each chromosome. The genes located on duplicated chromosomal segments are framed by same colors and connected by *same color lines* between the two relevant chromosomes. The tandem repeated genes are framed by red color on the chromosomes.

To obtain a global view about the distribution of C2H2-ZFP genes on the *B*. *rapa* genome, we retrieved the chromosomal location data of each identified C2H2-ZFP gene from the BRAD database (S1 Table) and constructed a physical map of C2H2-ZFP genes in *B*. *rapa* (Fig 3). Our results showed that 295 out of 301 (98.0%) *B*. *rapa* C2H2-ZFP genes were mapped into the 10 chromosomes (Fig 3), while the remaining six were located actually on scaffolds, and not mapped to any specific chromosome (S1 Table). As illustrated by Fig 3, these C2H2-ZFP genes were unequally distributed across the 10 chromosomes of *B*. *rapa*. The number of C2H2-ZF protein genes is 24, 42, 42, 10, 24, 30, 35, 17, 35 and 26 for A01, A02, A03, A04, A05, A06, A07, A08, A09 and A10, respectively. There existed an obvious tendency of clustering of C2H2-ZFP genes on some regions of *B*. *rapa* genome. 183 out of 301 (60.8%) C2H2-ZFP genes were involved in segmental duplication, and 16 pairs of C2H2-ZFP genes were involved in tandem duplication (Fig 3).

### Syntenic relationships between *B*. *rapa* and *A*. *thaliana*

*Brassica* species diverged from a common ancestor with the *Arabidopsis* lineage ~17 million years ago (MYA), and have all undergone a whole genome triplication (WGT) event ~15.9 MYA after their divergence from *Arabidopsis* [59–61]. *B*. *rapa* is a paleohexaploid and contains three subgenomes designated as least fractionalized (LF), moderately fractionized (MF1) and most fractionized (MF2) according to the extent of gene loss [51,60,61]. For each *B*. *rapa* C2H2-ZFP gene, we identified its syntenic paralogs on three subgenomes of *B*. *rapa* as well as its orthologs in *A*. *thaliana* from BRAD database (http://brassicadb.org/brad/). On the other hand, for each known *Arabidopsis* C2H2-ZFP gene, we checked if there existed the corresponding orthologous C2H2-ZFP genes on three *B*. *rapa* subgenomes. The syntenic relationships between the C2H2-ZFP genes of *B*. *rapa* and *A*. *thaliana* were summarized in S3 Table. All the 301 *B*. *rapa* C2H2-ZFP genes were assigned to one of the three subgenomes: 130 out of 301 (43.19%) on LF, 104 out of 301 (34.55%) on MF1, and 67 out of 301 (22.26%) on MF2. In 20 cases, the three copies were well conserved on the three subgenomes, while in 56 cases, two of the three copies were present, and in 112 cases, only one copy was present. 263 out of 301 (87.37%) *B*. *rapa* C2H2-ZFP genes found to their corresponding 164 syntenic orthologs in *A*. *thaliana*, which were derived from 23 blocks of 7 ancestral chromosomes of translocation Proto-Calepineae Karyotype (tPCK) [51,52,62,63]. According to the actual version of BRAD database, 38 out of 301 (12.63%) *B*. *rapa* C2H2-ZFP genes didn’t find their corresponding syntenic orthologs in *A*. *thaliana* (S3 Table), while 32 out of 196 (16.33%) *A*. *thaliana* C2H2-ZFP genes didn’t find their corresponding orthologs in *B*. *rapa*.

To have an idea about the selective pressure of these *B*. *rapa* C2H2-ZFP genes, the Ka, Ks and Ka/Ks values, as well as the divergence times were calculated for the different *A*. *thaliana* and *B*. *rapa* orthologous C2H2-ZFP gene pairs and summarized in S4 Table. The Ka/Ks ratios varied from 0.0116 to 1.4919, with an average value of 0.3082. Two orthologous gene pairs, AT1G04445-Bra030571 and AT5G61470-Bra029315, have Ka/Ks ratios (1.0174 and 1.4919, respectively) superior to 1, implying that they experienced positive selection, while all other 262 gene pairs have Ka/Ks ratios inferior to 1, implying that they experienced negative selection in the process of species evolution. The estimated divergence times for orthologous gene pairs between *A*. *thaliana* and *B*. *rapa* C2H2-ZFP genes ranged from 6.23 to 38.60 MY, with an average value of 18.29 MY, signifying that these C2H2-ZFP genes had evolved with different rates during species evolution [64].

### Additional domain analysis outside of the C2H2-ZF domain

In order to better classify the identified 301 *B*. *rapa* C2H2-ZFPs, we inspected the full-length protein sequence of each C2H2-ZFP by Smart tool analysis for additional known domain besides the C2H2-ZF domains. A total of 57 other known domains outside the C2H2-ZF domains were identified. Based on the presence or not of these additional domains and their organization, the 301 *B*. *rapa* C2H2-ZNPs were classified into 34 major groups and subgroups (S5 Table). The first major group includes 205 members (205/301 = 68.1%) containing only C2H2-ZF domains with no any other additional domain detected. The second major group includes 15 members (15/301 = 4.98%) containing each a Coiled-coil domain in addition to the C2H2-ZF domain(s). While the rest of 32 groups includes, 1–10 members with 1–7 additional known domains predicted to be involved in various molecular functions, such as protein-protein interaction (ANK, Ald_Xan_dh_C2, coiled-coil, PWWP, JmjC), protein binding (SET, PUG, CactinC_cactus, UBX), nucleic acid binding (KH, Ald_Xan_Dh_C2, tRNA_anti-codon, G_patch, DEXDc, tRNA-synt_2, HELICc, PPR, HA2, Kin17 curved), Histone binding/phosphorylation/methylation (POST SET, JmjN VEFS-Box, zf-TRM13_CCCH, MTS, Inositol_P domain), and Zinc ion binding (RING, AN1, IBR, Znf UBA, Znf C4HC3), *etc*. Other known domains such as C1, Transmembrane, NYN, P4HC, UFD1, zf-LYAR domains were also found to be associated with the C2H2-ZFP domain. All these additional known domains were also found in other plant species including *A*. *thaliana*, *Medicago truncatula*, *Nicotiana tobacum*, *Oryza sativa*, *Vitis vinifera*, *Glycine max*, *Glycine Soja*, *Solanum lycopersicum*, *Malus Domestica*, *Zea mays*, *etc*.

### Expression analysis of *B*. *rapa* C2H2-ZFP genes in different tissues

**Fig 4:**
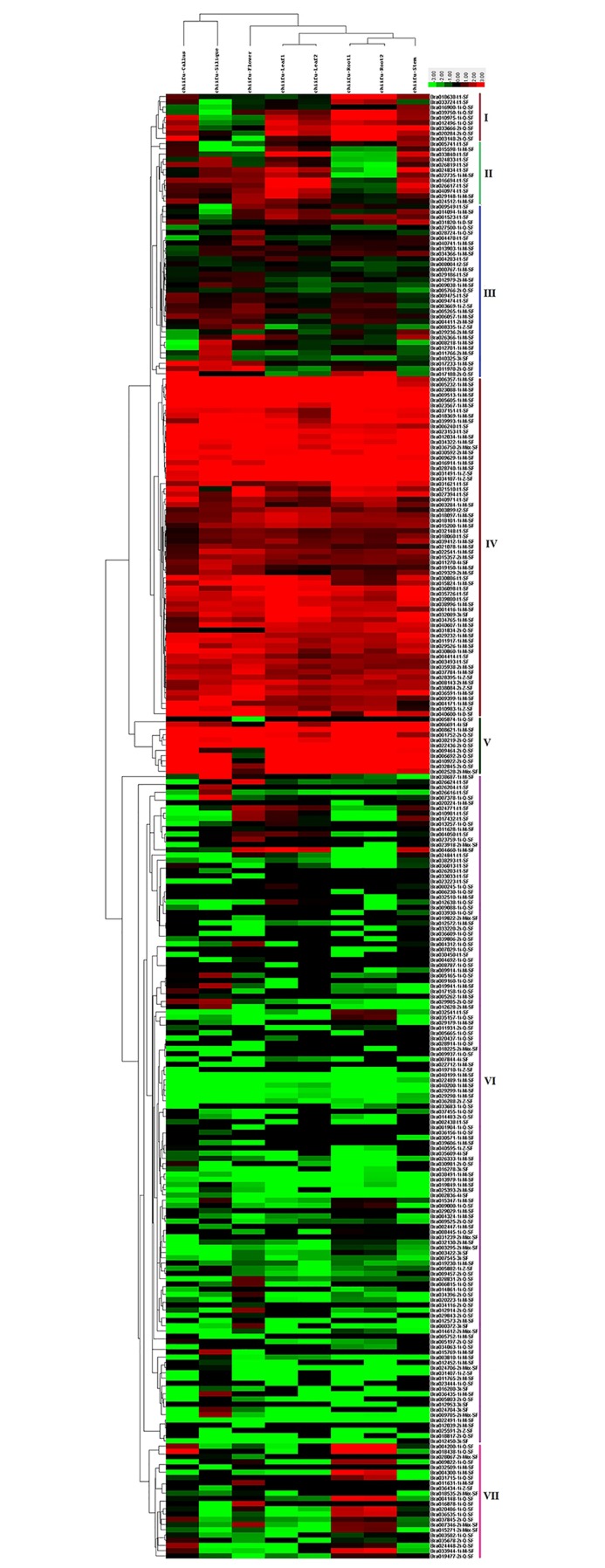
Expression profiles of 280 *Brassica rapa* C2H2-ZFP genes in different tissues or organs revealed by clustering analysis of RNA-seq data. The 280 genes were divided into seven major groups (I-VII) based on the log2-transformed fragments per kilobase of transcript per million fragments mapped (FPKM) values. The scale bar indicates relative expression level as shown on the top left side. The different tissue types are shown on the top side. The individual gene names are indicated on the right side.

To gain insights into the expression patterns of individual *B*. *rapa* C2H2-ZFP genes in different tissues, we used a publicly available RNA-seq transcriptomic dataset generated by deep sequencing of mRNA from six different tissues (callus, root, stem, leaf, flower and silique) of *B*. *rapa* (GSE43245). Except 17 genes, the expression data of 284 *B*. *rapa* C2H2-ZFP genes were available from the dataset, of which four genes showed an expression value of zero for all the six tissues and were then excluded from the analysis, while the remaining 280 genes were expressed in at least one of the six tissues. Based on the log2-transformed fragments per kilobase of transcript per million fragments mapped (FPKM) values of the dataset, a clustered heat map (Fig 4) was generated to displaying the expression patterns of *B*. *rapa* C2H2-ZF protein genes in callus, root (root_1 and root_2), stem, leaf (leaf_1 and leaf_2), flower and silique. The 280 *B*. *rapa* C2H2-ZFP genes were clustered into seven groups, The group I includes 9 genes, of which 88.8% were preferentially expressed both in root and stem. The group II includes 12 genes, which were all preferentially expressed in the stem, 83% in leaf, 66% in silique and 58% in flower. The group III includes 33 C2H2-ZFP genes, of which 72% were preferentially expressed in flower. The group IV includes 65 genes which were almost all highly and consecutively expressed in all the six tissues. The group V includes 11 genes, of which 100% were highly expressed in five tissues except in flower (54%). The group VI includes 128 genes, which were almost all very lowly (or not) expressed in all the tested tissues, except 31(24%) genes which were preferentially expressed in one or more tissues with relatively higher expression levels. The group VII includes 22 C2H2-ZFP genes, of which more than 63% were preferentially expressed in the root.

Table 2 summarized the distribution of 280 C2H2-ZFP genes in different expression groups of Fig 4 in relation to their classification by C2H2-ZF domain types in Fig 1. We can observe that the Br-t1-SF members were mainly distributed into the expression group VI (33.3%) and IV (31.2%), while the Br-1i-Q-SF into group VI (65.9%) and VII (19.1%), the Br-2i-Q-SF into group VI (47.1%) and V (20.6%), the Br-1i-M-SF into group IV (38.2%) and VI (38.2%), *etc*.

**Table 2:** Distribution of 280 *Brassica rapa* C2H2-ZFP genes in the different expression groups of Fig 4 in relation to the classification of their encoded proteins in Fig 1. Values in parentheses indicate the percentages of genes per total genes of each C2H2 finger type in each expression group.

Expression group	No. of genes	Br-t1-SF	Br-t2-SF	Br-1i-Q-SF	Br-2i-Q-SF	Br-1i-M-SF	Br-2i-M-SF	Br-1i-D-SF	Br-1i-Z-SF	Br-2i-Z-SF	Br-2i-Mix-SF	Br-3i-SF	Br-4i-SF
I	9	2 (4.1)^b^	-	4 (8.5)	3 (8.8)	-	-	-	-	-	-	-	-
II	12	8 (16.7)	-	-	-	4 (4.5)	-	-	-	-	-	-	-
III	33	7 (14.6)	1 (50.0)	2 (4.2)	3 (8.8)	12 (13.5)	4 (28.6)	1 (50.0)	2 (18.2)	-	-	1 (10.0)	-
IV	65	15 (31.2)	1 (50.0)	-	1 (2.9)	34 (38.2)	5 (35.7)	1 (50.0)	4 (36.4)	1 (25.0)	1 (7.1)	1 (10.0)	1 (20.0)
V	11	-	-	1 (2.1)	7 (20.6)	1 (1.1)	-	-	-	-	1 (7.1)	-	1 (20.0)
VI	128	16 (33.3)	-	31 (65.9)	16 (47.1)	34 (38.2)	5 (35.7)	-	4 (36.4)	3 (75.0)	8 (57.1)	8 (80.0)	3 (60.0)
VII	22	-	-	9 (19.1)	4 (11.8)	4 (4.5)	-	-	1 (9.1)	-	4 (28.6)	-	-
Total No.	280	48	2	47	34	89	14	2	11	4	14	10	5

### Expression analysis of the *B*. *rapa* C2H2-ZFP genes under abiotic stresses

**Fig 5:**
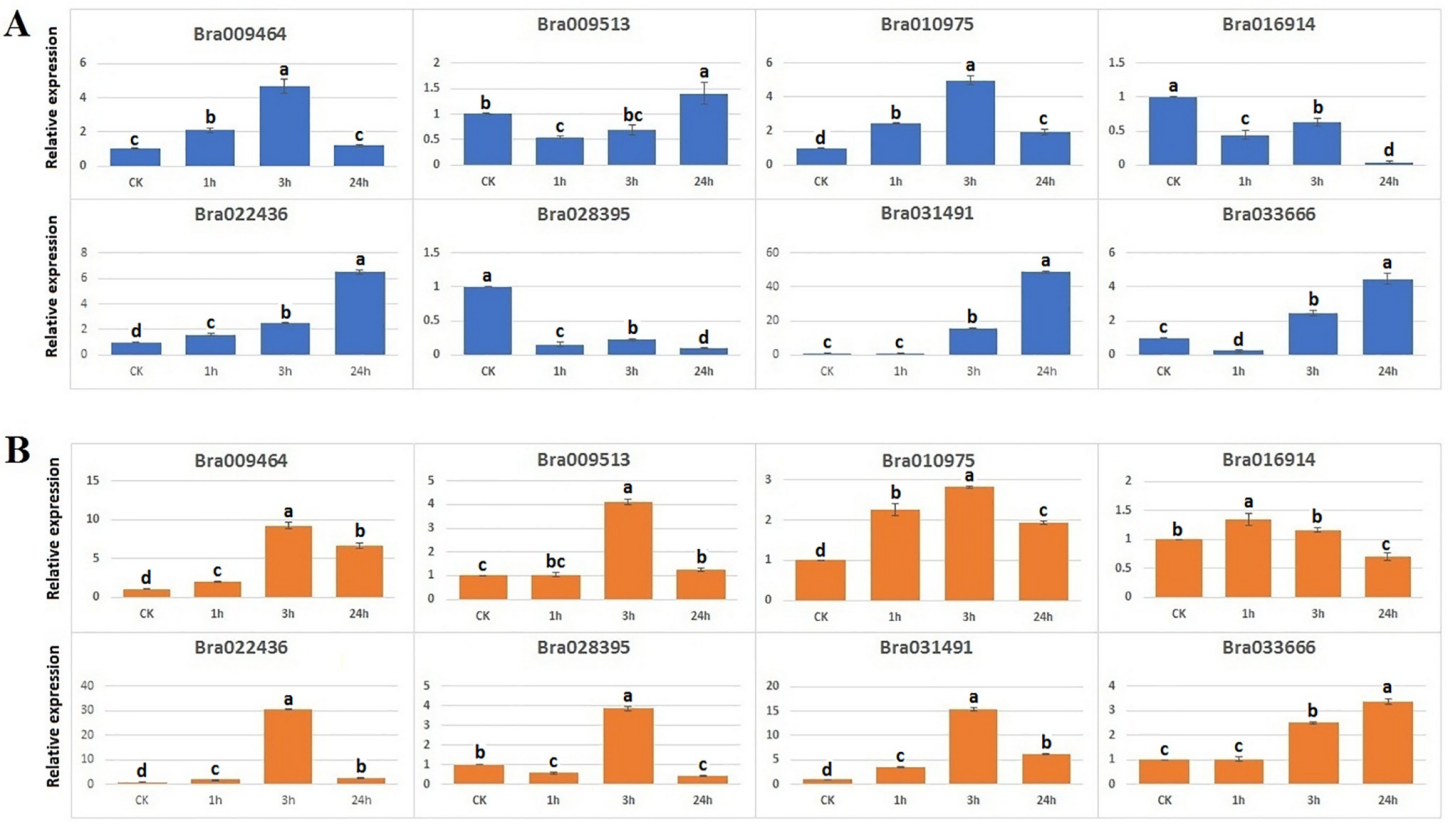
qRT-PCR expression patterns of eight *Brassica rapa* C2H2-ZFP genes under salt (A) and drought (B) treatments. The time points represent by x-axis and the scale of relative expression shown by y-axis. Tukey tests were used to determine differences among effects on different time courses under salt (A) and drought (B) treatments and different letters indicate the statistical significant deference’s with p ≤ 0.05.

To gain some information about the response of C2H2-ZFP genes to abiotic stresses, we examined the expression response of eight *B*. *rapa* C2H2-ZFP genes (S6 Table) to salt (200 mM NaCl) (Fig 5A) and drought (20% (w/v) PEG6000) (Fig 5B) stresses in the leaves of three weeks seedlings by using qRT-PCR technique. The eight genes were selected based on their high expression levels in most of the tested tissues. The results showed that all the eight *B*. *rapa* C2H2-ZFP genes were responsive to the two abiotic stress treatments.

In the case of salt treatment, six *B*. *rapa* C2H2-ZF protein genes were up-regulated and two down-regulated compared to control (CK) after 1 h, 3 h or 24 h of salt treatment, respectively (Fig 5A). The most remarkable case is the gene Bra031491 (corresponding to AT1G60640) which was induced by more than 40-fold under salt treatment at 24 h. Furthermore, the gene Bra022436 was progressively inducted along with the time and reached 6-fold at 24 h compared to the control.

In the case of drought treatment, all the six *B*. *rapa* C2H2-ZFP genes were responsive to the treatment compared to control (CK) after 1 h, 3 h or 24 h of treatment, respectively (Fig 5B). The highest induction was recorded for Bra022436, (corresponding to AT3G19580) which showed >30-fold up-regulation at 3h and moderately expressed at 24 h compared to the control. Furthermore, the genes Bra009464, Bra009513, Bra010975, and Bra031491 were progressively inducted along with the time and reached 2.5 to 15-fold at 3 h compared to the control and retained the expression more than 1 to 7-fold at 24 hours of treatment compared to the control.

### Comparison between *A*. *thaliana* and *B*. *rapa* orthologous C2H2-ZFP genes

In order to gain more insights into the degree of conservation between *B*. *rapa* and *A*. *thaliana* C2H2-ZFP genes, we compared the subcellular localization, number of C2H2 domain(s), number and type of additional domain(s), and expression pattern between their corresponding orthologs (S7 and S8 Tables). Our results showed that the subcellular localizations were globally well conserved for all of the 264 *A*. *thaliana*—*B*. *rapa* orthologous gene pairs, with some differences observed for 40 out of 264 (15.2%) cases where one or two additional localization(s) were detected for one of the two members. In 23 out of 264 (8.7%) cases, the number of C2H2-ZF domains contained by the compared orthologous C2H2-ZFPs was different between *B*. *rapa* and *A*. *thaliana*, with six cases of increase and 17 cases of decrease in the number of C2H2-ZF domains in *B*. *rapa* compared to *A*. *thaliana*. In 20 out of 264 (7.6%) cases, the type or/and number of additional known domains differed between *B*. *rapa* and *A*. *thaliana* (S7 Table).

**Fig 6:**
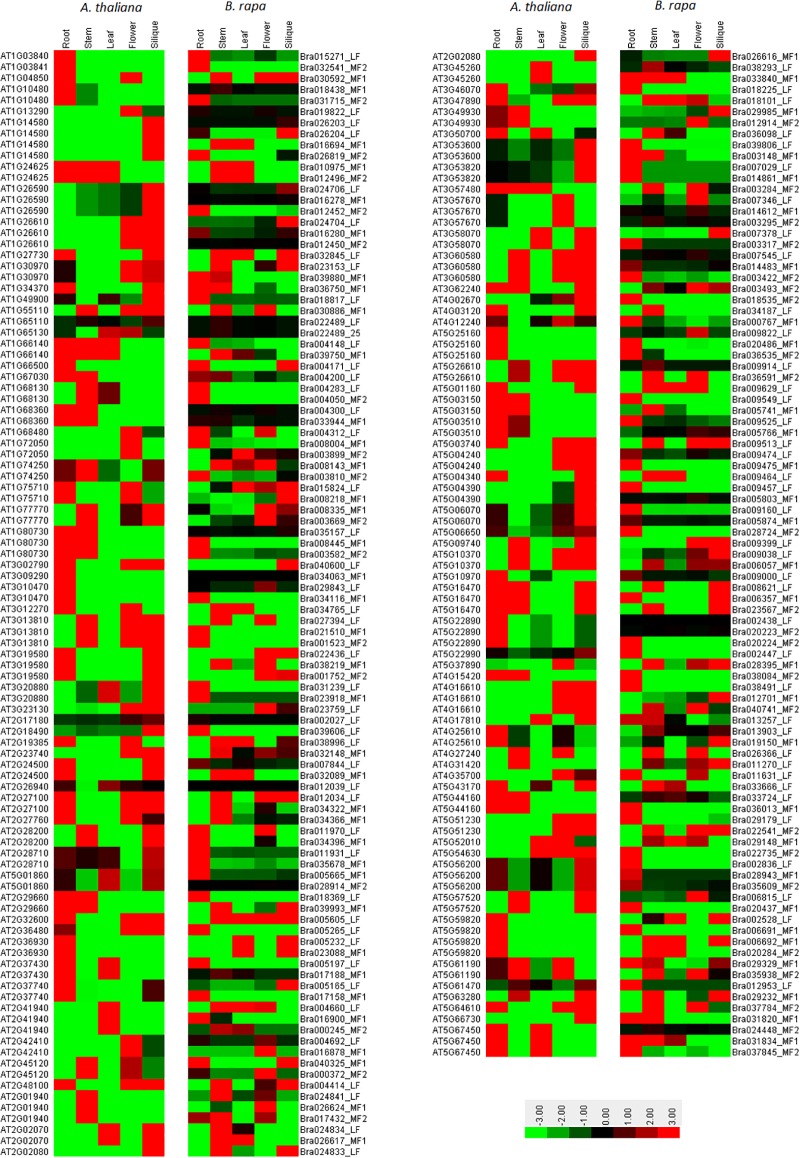
Heatmap comparison of expression patterns in five tissues or organs (root, stem, leaf, flower and silique) between *Brassica rapa* and *Arabidopsis thaliana* orthologous C2H2-ZFP genes (191 gene pairs). The map was constructed using Cluster software v3.0 (http://bonsai.hgc.jp/mdehoon/software/cluster/) with the option of “center genes”. The expression data of *B*. *rapa* C2H2-ZFP genes were extracted from the dataset GSE43245 (accession number) of NCBI Gene Expression Omnibus (GEO) database, while those of *A*. *thaliana* C2H2-ZFP genes were extracted from TAIR database (https://www.arabidopsis.org/) using the Arabidopsis eFP Browser. The *A*. *thaliana* gene names are indicated on the left side, while the *B*. *rapa* gene names (followed by their subgenome localisation) are indicated on the right side of the map. The scale representing the relative signal values is shown on the lower right side. The detail values based on which the map was constructed are summarized in S8 Table.

For comparison of expression pattern, we resembled the expression data of five tissues or organs (root, stem, leaf, flower and silique) for 191 out of 264 *A*. *thaliana*–*B*. *rapa* orthologous C2H2-ZFP gene pairs, and summarized them in S8 Table. The Pearson’s correlation coefficient (*P*) of expression levels (sum of values from five tissues or organs) between *B*. *rapa* and *A*. *thaliana* orthologous C2H2-ZFP genes was calculated and equaled 0.68. In 32 out of 191 (16.8%) cases (gene pairs), the correlation coefficients of expression patterns (different expression levels in five tissues or organs) between *B*. *rapa* and *A*. *thaliana* orthologous genes were superior to 0.8. Based on the data resembled in S8 Table, an expression heatmap was generated and presented in Fig 6, giving a global view of expression patterns of 191 *B*. *rapa*—*A*. *thaliana* orthologous C2H2-ZFP gene pairs. We can observe that while some C2H2-ZFP gene members tend to conserve similar expression patterns between *B*. *rapa* and *A*. *thalian*, many other members showed different expression patterns across the two species.

## Discussion

C2H2-ZF transcription factor family are widely existing in all eukaryotic organisms and involved in many biological processes, including plant growth, development, hormone signaling, and stress response [4,18,22,33]. To date, C2H2-ZFP genes have been investigated in multi-plant species, such as *A*. *thaliana*, rice, foxtail millet, durum wheat, tobacco, and poplar [14,15,18–21]. In the present study, a total of 301 C2H2-ZFP genes were identified with 533 C2H2-ZF domains in *B*. *rapa* genomes. The 301 C2H2-ZFPs were further subdivided into 12 different classes (Fig 1), based on the variation of the plant-specific conserved amino acid sequence “QALGGH” and distances between metal ligands within C2H2-ZF domains (Table 1). This classification was well supported by phylogenetic analysis with only a few exceptions (Fig 2). Majority of *B*. *rapa* C2H2-ZFPs contain one or more C2H2 domains with the QALGGH motif, and were identified as Q-type C2H2-ZFPs (Fig 1, S2 Table) [65]. The Q-type C2H2-ZFPs are plant specific, and have not been reported in any other organisms suggesting that these proteins may be involved in plant-specific life processing [8]. The complexity of these C2H2-ZF domains (Fig 1, S2 Table) plus the presence of additional known domain(s) in some C2H2-ZFP members (S5 Table) implies the high functional diversity of these C2H2-ZFPs in plant growth and development. Our classifications of *B*. *rapa* C2H2-ZFPs provide then helpful information for further functional characterization of this gene family among *Brassica* species.

It has been reported, that the model plant *A*. *thaliana*, has experienced three WGD events: a γ event shared with most dicots and two following genome duplications (α and β) shared with other associates of Brassicales [66]. *B*. *rapa* shares a common ancestor with *A*. *thaliana*, and has also experienced the three WGD events. In addition, the ancestor of *B*. *rapa* underwent an additional WGT event around 13 to 17 MYA followed by genome diploidization involving substantial genome reshuffling and gene losses in duplicated genomic blocks [47,67]. In the current study, the number of C2H2-ZFPs identified in *B*. *rapa* (301) was 1.54 times of that in *A*. *thaliana* (196), and 264 syntenic C2H2-ZFP orthologous gene pairs involving 148 *A*. *thaliana* C2H2-ZFP genes were identified between the two species (S3 and S4 Tables), showing the important gene losses of C2H2-ZFPs in *B*. *rapa* genome following the WGT event. Our syntenic relationship analysis (S3 Table) showed that in 20 cases, the three triplicated C2H2-ZFP gene copies were well conserved on the three subgenomes, while in 56 other cases, two of the three copies were maintained in *B*. *rapa* genome. Thus, about one-third of C2H2-ZFP gene members were increased due to the segmental chromosomal duplication caused by the WGT event. The tandem duplication is another central mode of gene expansion. For example, 1135, 1569, 1751 and 2137 tandemly duplicated gene clusters were discovered in *Thellungiella parvula*, *A*. *thaliana*, *B*. *rapa*, and *Arabidopsis lyrata*, respectively [68]. Among the 301 *B*. *rapa* C2H2-ZFP genes, 16 tandem arrays involving 32 C2H2-ZFP genes were identified (Fig 3), indicating that the tandem duplication has also contributed to the expansion of C2H2-ZFP gene family in the *B*. *rapa* genome. These duplicated C2H2-ZFP gene members are the results of natural and artificial selections during species evolution, and constitute then interesting candidates for studying the functional diversification of duplicated genes in *Brassica* crops [69, 70]

Our analysis of Ka/Ks values of the 264 orthologous C2H2-ZFP gene pairs between *A*. *thaliana* and *B*. *rapa* (S4 Table) indicated that almost all tested gene pairs (except AT1G04445-Bra030571 and AT5G61470-Bra029315) have the Ka/Ks ratios inferior to 1, implying that these genes have experienced purifying selection during species evolution [71,72]. AT1G04445 and AT5G61470 each encodes a putative protein of 172 aa and 304 aa in size, respectively, and have not been investigated in the model plant *A*. *thaliana*, constituting then the interesting targets together with their *B*. *rapa* orthologs for further functional study in both species. The estimated divergence time of each of the 264 orthologous C2H2-ZFP gene pairs between *A*. *thaliana* and *B*. *rapa* ranged from 6.23 to 38.60 MY, with an average value of 18.29 MY, showing that these gene members have undergone different selective pressures resulting in different evolutionary rates during species evolution [64]. The most slowly evolved gene pair is AT3G23130-Bra023759 with an estimated divergence time of 6.23 MY and a Ka/Ks ratio of 0.5723, while the most rapidly evolved gene pair is AT5G16470-Bra023567 with an estimated divergence time of 38.60 MY and a Ka/Ks ratio of 0.0115 (S4 Table). AT3G23130 (also named as *FLO10*, *FON1* or *SUPERMAN*) is characterized as a flower-specific gene controlling the boundary of the stamen and carpel whorls (https://www.arabidopsis.org/servlets/TairObject?id=37697&type=locus), while AT5G16470 (also named as MBS2) is characterized as required for induction of singlet oxygen-dependent gene expression and involved in ROS signaling mediated stress responsive pathway in *A*. *thaliana* (https://www.arabidopsis.org/servlets/TairObject?id=134268&type=locus). We can expect that those gene pairs with a low estimated divergence time value may maintain a conserved function between the two species, while those with a high divergence time value may generate new functions as have been suggested in other similar studies [73–75].

Many previous studies reported that most of C2H2-ZFPs function as transcription factors and play important roles in plant growth and development [23,36,37,40,76]. Our subcellular localization prediction showed that 92% of *B*. *rapa* C2H2-ZFPs were located in nucleus (S1 Table), which supported their functional roles as transcription factors in nucleus. Our expression pattern analysis showed that 280 of 301 *B*. *rapa* C2H2-ZFP genes were expressed in at least one of the six tested tissues, of which about one-third was almost always highly and consecutively expressed in all the tested tissues, one-third preferentially expressed in one to three tissues, and one-third very lowly expressed in all the tested tissues, illustrating the functional diversity of this gene family in *B*. *rapa* plant growth and development (Fig 4). Several members displaying tissue-specifically expressed patterns may be the good candidates for further in-depth studies for their molecular functions and potential applications on genetic improvement of *Brassica* species.

Our expression pattern analysis of eight *B*. *rapa* C2H2-ZFP genes in response to salt (Fig 5A) and drought (Fig 5B) stresses showed that all of them were responsive to the treatments of two abiotic stresses with different expression levels (Fig 5). One of the eight tested genes, Bra009464, orthologous to *A*. *thaliana* gene AT5G04340 or AtZAT6, was significantly up-regulated at 1h (1-fold) and 3h (up to 4-fold) under salt stress (Fig 5A). Interestingly, AtZAT6 was also up-regulated at 1h and 3h after treatment of Col-0 plants by 300 mM NaCl as demonstrated in a previous study [77], suggesting that the functions of two orthologous genes were probably well conserved in both species. Moreover, overexpression of AtZAT6 positively regulated the cadmium tolerance in *Arabidopsis* through the glutathione-dependent pathway [78], and showed pleiotropic phenotypes with curly leaves, small sized plant at vegetative stage and reduced size of floral organs and siliques at the reproductive stage [77]. Similarly, the orthologous gene of Bra009513 in *A*. *thaliana* is AT5G03740 or HD2C, which was shown to be involved in ABA, salt [79], and heat stress responses [80]. The orthologous gene of Bra022436 in *A*. *thaliana* is AT3G19580 or AZF2, which was shown to function as a transcriptional repressor involved in the inhibition of plant growth under abiotic stress conditions [24,81]. These results indicated that all the eight tested genes were more or less involved in stress response in *B*. *rapa*, in a similar way as their orthologs in *A*. *thaliana*. Further stress responsive expression analysis of a larger number of *B*. *rapa* C2H2-ZFP genes, including those lowly expressed ones, would allow to identify the most actives members involved in stress tolerance in *Brassica* crops.

Our comparative analysis between *A*. *thaliana* and *B*. *rapa* corresponding orthologous C2H2-ZFP genes (264 gene pairs) showed that the majority of them encode proteins with conserved subcellular localization, C2H2-ZF domain(s) and additional known domain(s) between the two species (S7 Table). This means that most of the C2H2-ZFP members may conserved their basic cellular functions across *Arabidopsis* and *Brassica* species, while the other minor portion (7~15%) of C2H2-ZFP genes may have deviated from their initial functions or gained new functions during the evolution of plant species. Comparison of expression pattern between *A*. *thaliana* and *B*. *rapa* corresponding orthologous C2H2-ZFP genes (191 gene pairs) showed that there existed a good positive correlation of expression levels (sum of values from five tissues or organs) between *Arabidopsis* and *B*. *rapa* C2H2-ZFP genes, with a *P* value equals 0.68 (S8 Table), reflecting somewhat the degree of functional conservation of these C2H2-ZFP genes between the two species. However, only about one-third of *A*. *thaliana*—*B*. *rapa* orthologous C2H2-ZFP gene pairs displayed *P* values ranging from 0.5 to 1 for their expression patterns in five tissues or organs, while other two-third genes pairs displayed *P* values ranging from -1 to 0.5 (S8 Table), suggesting that the functional roles in plant growth and development may be modified between the two species for most of these C2H2-ZFP genes by differed expression patterns which were well illustrated in Fig 6.

## Conclusions

In the current study, we analyzed the C2H2-ZFP family in *B*. *rapa* using the available *Brassica* genome databases. A total of 301 C2H2-ZFP genes were identified and further characterized through analysis of conserved amino acid residues in C2H2-ZF domains and their organization, subcellular localization, phylogeny, additional domain, chromosomal location, synteny relationship, and expression pattern, *etc*. We also analyzed the expression patterns of eight *B*. *rapa* C2H2-ZFP genes under salt and drought stress conditions by using qRT-PCR technique. Our results showed that about one-third of these *B*. *rapa* C2H2-ZFP genes were originated from segmental duplication caused by the WGT around 13 to 17 MYA, one-third of them were highly and consecutively expressed in all tested tissues, and 92% of them were located in nucleus by prediction supporting then their functional roles as transcription factors, of which some may play important roles in plant growth and development. In addition, a few of these *B*. *rapa* C2H2-ZFP genes were shown to be involved in stress responses in a similar way as their orthologs in *A*. *thaliana*. Comparison between *A*. *thaliana* and *B*. *rapa* orthologous C2H2-ZFP genes showed that most of these C2H2-ZFP gene members encodes proteins with conserved subcellular localization and functional domains between the two species but differed in their expression patterns in five tissues or organs. Thus, our study provides valuable information for further functional determination of each C2H2-ZFP gene across the *Brassica* species, and may help to select the appropriate gene targets for further genetic engineering and genetic improvement of *Brassica* crops.

## Supporting information

S1 Fig Multiple sequence alignments of all the identified 534 C2H2-ZFs in *Brassica rapa*. (PDF)

S1 Table List of 301 *Brassica rapa* C2H2-ZFP genes and their related information. (XLSX)

S2 Table Classification of the 301 *Brassica rapa* C2H2-ZFPs according to the organization of their contained C2H2 fingers. (XLSX)

S3 Table Synteny relationships between *Arabidopsis thaliana* and *Brassica rapa* C2H2-ZFP genes. (XLSX)

S4 Table Ka, Ks, Ka/Ks ratio and divergence time between *Arabidopsis thaliana* and *Brassica rapa* corresponding orthologous C2H2-ZFP genes (264 gene pairs). (XLSX)

S5 Table Classification of *Brassica rapa* C2H2-ZFPs based on the presence and organization of additional domain(s). (XLSX)

S6 Table The information of primers used for quantitative real-time PCR (qRT-PCR). (XLSX)

S7 Table Comparison of the subcellular localization, number of C2H2 domains, and number and type of additional domains between *Arabidopsis thaliana* and *Brassica rapa* corresponding orthologous C2H2-ZFP genes (264 gene pairs). (XLSX)

S8 Table Comparison of expression profiles (values) in root, stem, leaf, flower and silique, and their Pearson’s correlation coefficients between *Brassica rapa* and *Arabidopsis thaliana* orthologous C2H2-ZFP genes (191 gene pairs). (XLSX)
