# Supplementary material for: Comprehensive genomic survey, structural classification and expression analysis of C2H2 zinc finger protein gene family in Brassica rapa L
Source: PLoS One. 2019 May 6;14(5):e0216071. doi: 10.1371/journal.pone.0216071 (PMC6502316; doi:10.1371/journal.pone.0216071)
Supplement: S1 Fig — (PDF) [file pone.0216071.s001.pdf]

S1 Fig. Multiple sequence alignment of 534 C2H2 domains of *B. rapa*.

```
               1        10        20        30        40        50
               ....|....|....|....|....|....|....|....|....|....|....|.
Bra000245-Q    FECHY--C---------F-R--K-------------FP-TSQALGGHK--N-A--H
Bra000372-Q-b  FKCET--C---------G-K--V-------------FN-SYQALGGHR--A-S--H
Bra000372-Q-c  HQCPI--C---------F-R--V-------------FT-SGQALGGHK--R-S--H
Bra001026-Q-b  HRCRL--C---------E-R--V-------------FS-TYQALGGHQ--T-F--H
Bra001318-Q    YTCSF--C---------V-R--G-------------FS-NAQALGGHM--N-I--H
Bra001752-Q-a  YNCSV--C---------G-K--A-------------FL-SYQALGGHK--A-S--H
Bra001752-Q-b  HECSI--C---------H-K--V-------------FP-TGQALGGHK--R-C--H
Bra001904-Q    YDCDI--C---------M-R--G-------------FT-SPQALGGHR--N-I--H
Bra002027-Q-b  HRCDI--C---------S-K--A-------------FS-SGQALGGHM--R-R--H
Bra002528-Q-b  HPCPI--C---------G-V--E-------------FP-MGQALGGHM--R-R--H
Bra002836-Q-c  HVCVT--C---------N-K--S-------------FA-SYQALGGHR--A-S--H
Bra002836-Q-d  HVCNI--C---------Q-K--S-------------FT-TGQALGGHK--R-C--H
Bra003148-Q-a  FECKT--C---------N-R--K-------------FD-SFQALGGHR--A-S--H
Bra003148-Q-b  HKCSI--C---------G-Q--M-------------FG-TGQALGGHM--R-K--H
Bra003317-Q    FECHY--C---------F-R--N-------------FP-TSQALGGHQ--N-A--H
Bra003422-Q-b  FTCET--C---------G-K--V-------------FK-SYQALGGHR--A-S--H
Bra003422-Q-c  HECPI--C---------L-R--V-------------FA-SGQALGGHK--R-S--H
Bra003582-Q    FSCNY--C---------K-R--K-------------FY-SSQALGGHQ--N-A--H
Bra004148-Q    FSCNY--C---------Q-R--K-------------FY-SSQALGGHQ--N-A--H
Bra004200-Q    YECQY--C---------C-R--E-------------FA-NSQALGGHQ--N-A--H
Bra004312-Q    YECRF--C---------S-L--K-------------FC-KSQALGGHM--N-R--H
Bra004692-Q    YTCSF--C---------R-R--E-------------FR-SAQALGGHM--N-V--H
Bra005165-Q    YSCSF--C---------R-R--E-------------FK-SAQALGGHM--N-V--H
Bra005665-Q    FGCTF--C---------E-K--E-------------FS-TSQALGGHQ--N-A--H
Bra005197-Q-a  FECKT--C---------N-K--R-------------FS-SFQALGGHR--A-S--H
Bra005197-Q-b  HECSI--C---------S-Q--S-------------FG-TGQALGGHM--R-R--H
Bra005766-Q-a  YQCKT--C---------D-K--S-------------FH-SFQALGGHR--T-S--H
Bra005766-Q-b  HECSI--C---------K-A--E-------------FS-SGQALGGHM--R-R--H
Bra005803-Q-a  YQCKT--C---------D-R--T-------------FP-SFQALGGHR--A-S--H
Bra005803-Q-b  HECGI--C---------G-A--E-------------FT-SGQALGGHM--R-R--H
Bra005840-Q    YKCNY--C---------P-K--T-------------YS-KGQALGGHQ--N-V--H
Bra005874-Q    YTCSF--C---------G-R--E-------------FK-SAQALGGHM--N-V--H
Bra006230-Q    FPCQY--C---------P-R--K-------------FY-TSQALGGHQ--N-A--H
Bra006302-Q-b  HQCRL--C---------S-K--V-------------FS-SYQALGGHQ--T-S--H
Bra006691-Q-a  FTCKT--C---------L-K--E-------------FH-SFQALGGHR--A-S--H
Bra006691-Q-b  HPCPI--C---------G-V--E-------------FP-MGQALGGHM--R-R--H
Bra006691-Q-c  YQCKT--C---------D-R--T-------------FP-SFQALGGHR--A-S--H
Bra006691-Q-d  HECGI--C---------G-A--E-------------FT-SGQALGGHM--R-R--H
Bra006692-Q-a  FTCKT--C---------L-K--E-------------FH-SFQALGGHR--A-S--H
Bra006692-Q-b  HPCPI--C---------G-V--E-------------FP-MGQALGGHM--R-R--H
Bra006815-Q    FSCNY--C---------Q-R--K-------------FY-SSQALGGHQ--N-A--H
Bra007029-Q    YICDF--C---------E-R--G-------------FS-NAQALGGHM--N-I--H
Bra007378-Q    FECHY--C---------F-R--N-------------FP-TSQALGGHQ--N-A--H
Bra007545-Q-b  YTCET--C---------G-K--V-------------FK-SYQALGGHR--A-S--H
Bra007545-Q-c  HECPI--C---------L-R--V-------------FA-SGQALGGHK--R-S--H
Bra008445-Q    FSCNY--C---------Q-R--K-------------FY-SSQALGGHQ--N-A--H
Bra008787-Q    FPCQY--C---------P-R--K-------------FY-TSQALGGHQ--N-A--H
```

```
Bra009000-Q     FSCNY--C---------Q-R--T-------------FY-SSQALGGHQ--N-A--H
Bra009088-Q     YKCKY--C---------H-K--R-------------FS-KRQALGGHQ--N-A--H
Bra009160-Q     YSCSF--C---------G-R--E-------------FK-SAQALGGHM--N-V--H
Bra009457-Q-a   YQCKT--C---------D-R--T-------------FP-SFQALGGHR--A-S--H
Bra009457-Q-b   HECGV--C---------G-A--E-------------FT-SGQALGGHM--R-R--H
Bra009464-Q-a   HKCSV--C---------D-K--A-------------FS-SYQALGGHK--A-S--H
Bra009464-Q-b   HVCSI--C---------N-K--S-------------FA-TGQALGGHK--R-C--H
Bra009525-Q-a   YQCQT--C---------D-K--S-------------FH-SFQALGGHR--A-S--H
Bra009525-Q-b   HECSI--C---------K-A--E-------------FS-SGQALGGHM--R-R--H
Bra009822-Q     FSCNY--C---------Q-R--T-------------FY-SSQALGGHQ--N-A--H
Bra009937-Q     YTCQF--C---------S-K--G-------------FS-TTQALGGHQ--N-A--H
Bra010922-Q-a   YKCSV--C---------D-K--S-------------FS-SYQALGGHK--A-S--H
Bra010922-Q-b   HVCTI--C---------H-K--S-------------FP-SGQALGGHK--R-C--H
Bra010975-Q     FSCNY--C---------R-R--K-------------FY-SSQALGGHQ--N-A--H
Bra011601-Q-b   FECRG--C---------K-K--V-------------FG-SHQALGGHR--A-S--H
Bra011931-Q-a   FACKT--C---------N-K--E-------------FS-SFQALGGHR--A-S--H
Bra011931-Q-b   HECPI--C---------G-A--E-------------FA-VGQALGGHM--R-K--H
Bra011970-Q-a   YECKT--C---------N-R--T-------------FP-SFQALGGHR--A-S--H
Bra011970-Q-b   HECSI--C---------G-S--E-------------FT-SGQALGGHM--R-R--H
Bra012450-Q-c   HECPI--C---------F-R--V-------------FK-SGQALGGHK--R-S--H
Bra012496-Q     FSCNY--C---------R-R--K-------------FY-SSQALGGHQ--N-A--H
Bra012638-Q     YACNF--C---------R-R--E-------------FR-SAQALGGHM--N-V--H
Bra012914-Q-a   YKCSV--C---------G-K--S-------------FP-SYQALGGHK--T-S--H
Bra012914-Q-b   HKCSI--C---------F-K--S-------------FP-SGQALGGHK--R-C--H
Bra012953-Q-b   FTCDI--C---------G-K--V-------------LH-SYQALGGHR--T-S--H
Bra012953-Q-c   YKCEI--C---------G-R--V-------------FG-SGQALGGHK--K-V--H
Bra013257-Q     YTCNF--C---------R-R--E-------------FR-SAQALGGHM--N-V--H
Bra014483-Q-a   FTCET--C---------G-K--V-------------FK-SYQALGGHR--A-S--H
Bra014483-Q-b   HECPI--C---------L-R--V-------------FT-SGQALGGHK--R-S--H
Bra014861-Q     YICDF--C---------E-R--G-------------FS-NAQALGGHM--N-I--H
Bra016278-Q-c   HECPF--C---------F-R--V-------------FK-SGQALGGHK--R-S--H
Bra016280-Q-c   HECPI--C---------F-R--V-------------FK-SGQALGGHK--R-S--H
Bra016878-Q     YTCSF--C---------R-R--D-------------FR-SAQALGGHM--N-V--H
Bra016900-Q     FECHY--C---------F-R--N-------------FP-TSQALGGHQ--N-A--H
Bra017158-Q     YACSF--C---------R-R--E-------------FR-SAQALGGHM--N-V--H
Bra017188-Q-a   FECKT--C---------N-K--R-------------FS-SFQALGGHR--A-S--H
Bra017188-Q-b   HECTI--C---------G-Q--S-------------FG-TGQALGGHM--R-R--H
Bra017706-Q-b   FECGG--C---------K-K--V-------------FG-SHQALGGHR--A-S--H
Bra017706-Q-c   HKCNI--C---------F-R--V-------------FS-SGQALGGHM--R-C--H
Bra018225-Q-b   HRCPI--C---------G-V--E-------------FP-IGQALGGHM--R-K--H
Bra018438-Q     LECQY--C---------G-K--E-------------FA-NSQALGGHQ--N-A--H
Bra018817-Q-a   YKCDV--C---------G-K--E-------------FT-SYQALGGHK--A-S--H
Bra018817-Q-b   HKCSI--C---------N-V--V-------------FP-TGQALGGHK--R-R--H
Bra019476-Q-b   HRCSI--C---------G-L--E-------------FE-MGQALGGHM--R-K--H
Bra019477-Q-a   FRCKT--C---------M-K--E-------------FS-SFQALGGHR--A-S--H
Bra019477-Q-b   HTCPI--C---------G-L--E-------------FP-MGQALGGHM--R-K--H
Bra020284-Q-a   FTCKT--C---------L-K--E-------------FQ-SFQALGGHR--A-S--H
Bra020284-Q-b   HPCPI--C---------G-V--E-------------FP-MGQALGGHM--R-K--H
Bra020437-Q     FSCNY--C---------Q-R--K-------------FY-SSQALGGHQ--N-A--H
Bra020486-Q     FSCNY--C---------Q-R--T-------------FY-SSQALGGHQ--N-A--H
Bra020571-Q     YICHF--C---------N-K--R-------------FS-TSQALGGHQ--N-A--H
```

```
Bra022436-Q-a   HKCSV--C---------G-K--A-------------FP-SYQALGGHK--A-S--H
Bra022436-Q-b   HECSI--C---------H-K--V-------------FP-TGQALGGHK--R-C--H
Bra023444-Q     FPCQY--C---------P-R--K-------------FF-TSQALGGHQ--N-A--H
Bra023759-Q     YTCSF--C---------K-R--E-------------FR-SAQALGGHM--N-V--H
Bra024448-Q-a   FKCTV--C---------G-K--S-------------FN-SYQALGGHK--T-S--H
Bra024448-Q-b   HTCSI--C---------F-K--S-------------FS-SGQALGGHK--R-C--H
Bra024704-Q-c   HECPI--C---------F-R--V-------------FK-SGQALGGHK--R-S--H
Bra024706-Q-b   HECPV--C---------F-K--V-------------YS-SGQALGGHK--R-S--H
Bra027500-Q     YECTF--C---------K-R--G-------------FT-NAQALGGHM--N-I--H
Bra028724-Q     FKCHY--C---------S-R--K-------------FP-TSQALGGHQ--N-A--H
Bra028791-Q-a   YQCKT--C---------D-R--T-------------FS-SFQALGGHR--A-S--H
Bra028791-Q-b   HKCGI--C---------G-A--E-------------FT-SGQALGGHM--R-R--H
Bra028831-Q-a   YQCKT--C---------D-K--S-------------FH-SFQALGGHR--A-S--H
Bra028831-Q-b   HECSI--C---------K-A--E-------------FS-SGQALGGHM--R-R--H
Bra028914-Q     FACTF--C---------K-K--E-------------FS-TSQALGGHQ--N-A--H
Bra028943-Q-c   HVCVT--C---------N-K--S-------------FT-SYQALGGHR--A-S--H
Bra028943-Q-d   HVCDI--C---------H-K--S-------------FP-TGQALGGHK--R-R--H
Bra029315-Q-a   FICDV--C---------G-K--V-------------FG-SYQALGGHR--T-S--H
Bra029315-Q-b   HQCGV--C---------G-R--E-------------FE-SGQALGGHM--K-T--H
Bra029843-Q-a   YQCKT--C---------D-R--T-------------FP-SFQALGGHR--A-S--H
Bra029843-Q-b   HECGI--C---------G-A--E-------------FT-SGQALGGHM--R-R--H
Bra029985-Q-a   YKCSV--C---------G-K--S-------------FP-SYQALGGHK--T-S--H
Bra029985-Q-b   HKCSI--C---------F-K--S-------------FP-SGQALGGHK--R-C--H
Bra030981-Q-a   FECKT--C---------N-R--R-------------FS-SFQALGGHR--A-S--H
Bra030981-Q-b   HKCSR--C---------D-Q--S-------------FG-TGQALGGHM--R-R--H
Bra031715-Q     LECQY--C---------G-K--E-------------FA-NSQALGGHQ--N-A--H
Bra031834-Q-a   YKCTV--C---------G-K--S-------------FS-SYQALGGHK--T-S--H
Bra031834-Q-b   HTCSI--C---------F-K--S-------------FS-SGQALGGHK--R-C--H
Bra032661-Q-a   FQCKA--C---------K-K--V-------------FA-SHQALGGHR--A-S--H
Bra032661-Q-b   HECTV--C---------H-R--V-------------FS-SGQALGGHK--R-C--H
Bra032845-Q-a   YTCSV--C---------D-K--S-------------FS-SYQALGGHK--A-S--H
Bra032845-Q-b   HVCSI--C---------H-K--S-------------FP-SGQALGGHK--R-C--H
Bra033220-Q-a   FQCKA--C---------K-K--V-------------FT-SHQALGGHR--E-S--H
Bra033220-Q-b   HECTI--C---------H-R--V-------------FS-SGQALGGHK--R-C--H
Bra033666-Q-a   YKCGV--C---------D-K--V-------------FL-SYQALGGHK--A-S--H
Bra033666-Q-b   HVCSI--C---------H-K--S-------------FA-TGQALGGHK--R-C--H
Bra033683-Q     YECTF--C---------K-R--G-------------FT-NAQALGGHM--N-I--H
Bra033930-Q     YECRF--C---------S-L--K-------------FC-KSQALGGHM--N-R--H
Bra034063-Q     YLCSF--C---------V-R--G-------------FS-NAQALGGHM--N-I--H
Bra034116-Q-a   YQCKT--C---------D-R--T-------------FP-SFQALGGHR--A-S--H
Bra034116-Q-b   HECGI--C---------G-A--E-------------FT-SGQALGGHM--R-R--H
Bra034396-Q-a   YECKT--C---------S-R--T-------------FS-SFQALGGHR--A-S--H
Bra034396-Q-b   HECSI--C---------G-S--E-------------FT-SGQALGGHM--R-R--H
Bra035157-Q     FSCNY--C---------Q-R--K-------------FY-SSQALGGHQ--N-A--H
Bra035609-Q-c   HVCVT--C---------N-K--S-------------FT-SYQALGGHR--A-S--H
Bra035609-Q-d   HVCNK--C---------H-K--S-------------FP-TGQALGGHK--R-R--H
Bra035678-Q-a   FACKT--C---------N-K--E-------------FP-SFQALGGHR--A-S--H
Bra035678-Q-b   HECPI--C---------G-A--E-------------FA-VGQALGGHM--R-K--H
Bra036156-Q     FPCLF--C---------S-R--K-------------FH-SSQALGGHQ--N-A--H
Bra036535-Q     FSCNY--C---------Q-R--S-------------FY-SSQALGGHQ--N-A--H
Bra036609-Q     FSCPT--C---------K-K--G-------------FP-SSQALGGHQ--N-A--H
```

```
Bra037455-Q       FSCLF--C---------S-R--K-------------FQ-SSQALGGHQ--N-A--H
Bra037845-Q-a     YKCTV--C---------G-K--S-------------FS-SYQALGGHK--T-S--H
Bra037845-Q-b     HTCSI--C---------F-K--S-------------FS-SGQALGGHK--R-C--H
Bra038219-Q-a     YKCSV--C---------G-K--A-------------FP-SYQALGGHK--A-S--H
Bra038219-Q-b     HECSI--C---------R-K--V-------------FP-TGQALGGHK--R-C--H
Bra039750-Q       FSCNY--C---------Q-R--K-------------FY-SSQALGGHQ--N-A--H
Bra039806-Q-a     FECKT--C---------N-R--K-------------FD-SFQALGGHR--A-S--H
Bra039806-Q-b     HKCSI--C---------S-Q--M-------------FG-TGQALGGHI--R-R--H
Bra040325-Q-b     FKCET--C---------G-K--A-------------FK-SYQALGGHR--A-S--H
Bra040325-Q-c     HQCPI--C---------F-R--V-------------FS-SGQALGGHK--R-S--H
Bra000372-M1-a    YKCRF--C---------F-K--S-------------FL-NGRALGGHM--R-S--H
Bra002528-M1-a    FTCKT--C---------L-K--E-------------FL-SFQAWGGHR--A-S--H
Bra002836-M1-a    HICCE--C---------G-K--R-------------FK-SGKALGGHK--R-I--H
Bra003422-M1-a    YKCRF--C---------S-K--S-------------FF-NGRALGGHM--R-S--H
Bra004300-M1      YECQY--C---------C-R--E-------------FG-NSQALGGPQ--N-A--H
Bra004660-M1      FECHY--C---------F-R--N-------------FP-TSQALGGPQ--N-A--H
Bra007545-M1-a    YKCKF--C---------F-K--S-------------FV-NGRALGGHM--R-S--H
Bra011601-M1-c    HKCNI--C---------Y-R--V-------------YS-NGQALRGHM--R-Y--H
Bra012701-M1      HKCKL--C---------S-K--S-------------FC-NGRALGGHM--K-S--H
Bra012953-M1-a    RNCQV--C---------K-K--S-------------FP-NGRALGGHM--R-S--H
Bra018225-M1-a    YRCKT--C---------M-K--E-------------FS-SFQSLGGHR--A-S--H
Bra025393-M1-b    YRCKV--C---------G-K--S-------------FG-CFQSLGGHQ--N-L--H
Bra028943-M1-a    HMCSE--C---------G-K--Q-------------FM-SGKALGGHK--R-I--H
Bra033944-M1      YECQC--C---------C-R--E-------------FG-NSQALGGPQ--N-A--H
Bra035609-M1-a    HICCA--C---------G-K--S-------------FT-SGKALGGHK--S-L--H
Bra036608-M1      YICRF--C---------N-K--W-------------WP-TSQSLGGHM--N-S--H
Bra038491-M1      HKCKL--C---------S-K--S-------------FC-NGRALGGHM--K-S--H
Bra040325-M1-a    YKCRF--C---------F-K--S-------------FI-NGSALGGHM--R-S--H
Bra040741-M1      HKCKL--C---------S-K--S-------------FC-NGRALGGHM--K-S--H
Bra002836-M2-b    VDCCV--C---------H-K--K-------------FT-SMKALYGHM--R-F--H
Bra011601-M2-a    RPCTE--C---------G-K--T-------------FW-SWKALFGHM--R-C--H
Bra012039-M2-a    HFCVI--C---------S-K--Q-------------FS-SGKAYGGHV--R-I--H
Bra012450-M2-a    FVCKF--C---------N-K--R-------------FA-CGKSLGGHI--R-T--H
Bra012450-M2-b    LYCRE--C---------G-K--G-------------FP-SSKALCGHM--A-S--H
Bra012452-M2      YVCKF--C---------N-K--K-------------FT-SGKSLGGHI--R-I--H
Bra016280-M2-a    FVCRF--C---------S-K--R-------------FP-CGKSLGGHI--R-T--H
Bra016280-M2-b    LYCRE--C---------G-K--G-------------FP-SSKALCGHM--A-C--H
Bra017706-M2-a    RPCTE--C---------G-K--T-------------FW-SWTALFGHM--R-C--H
Bra019476-M2-a    FRCKT--C---------M-K--E-------------FS-SFHALGVHR--V-S--H
Bra022712-M2      HICDT--C---------R-R--Q-------------FP-TLKALYGHQ--R-V--H
Bra024704-M2-a    FVCKF--C---------S-K--R-------------FA-CGKSLGGHI--R-T--H
Bra024704-M2-b    LYCRE--C---------G-K--G-------------FT-SSKALCGHM--A-C--H
Bra024706-M2-a    FVCKF--C---------N-K--K-------------FP-SGKSLGGHI--R-I--H
Bra035609-M2-b    VDCCV--C---------H-K--K-------------FP-SMKALYGHM--R-F--H
Bra001523-M3-a    FVCEI--C---------N-K--G-------------FQ-RDQNLQLHK--R-G--H
Bra004050-M3-a    YVCEI--C---------N-Q--G-------------FQ-RDQNLQMHR--R-R--H
Bra004283-M3-a    YVCEI--C---------N-Q--G-------------FQ-RDQNLQMHR--R-R--H
Bra004414-M3-a    HRCVA--C---------F-K--M-------------FN-RRQHLVEHM--KIS--H
Bra004414-M3-c    RGCTL--C---------L-R--I-------------FE-DATALADHK--NKC--H
Bra005741-M3-a    FVCEI--C---------N-K--G-------------FQ-RDQNLQLHR--R-G--H
Bra009549-M3-a    FVCEI--C---------N-K--G-------------FQ-RDQNLQLHR--R-G--H
```

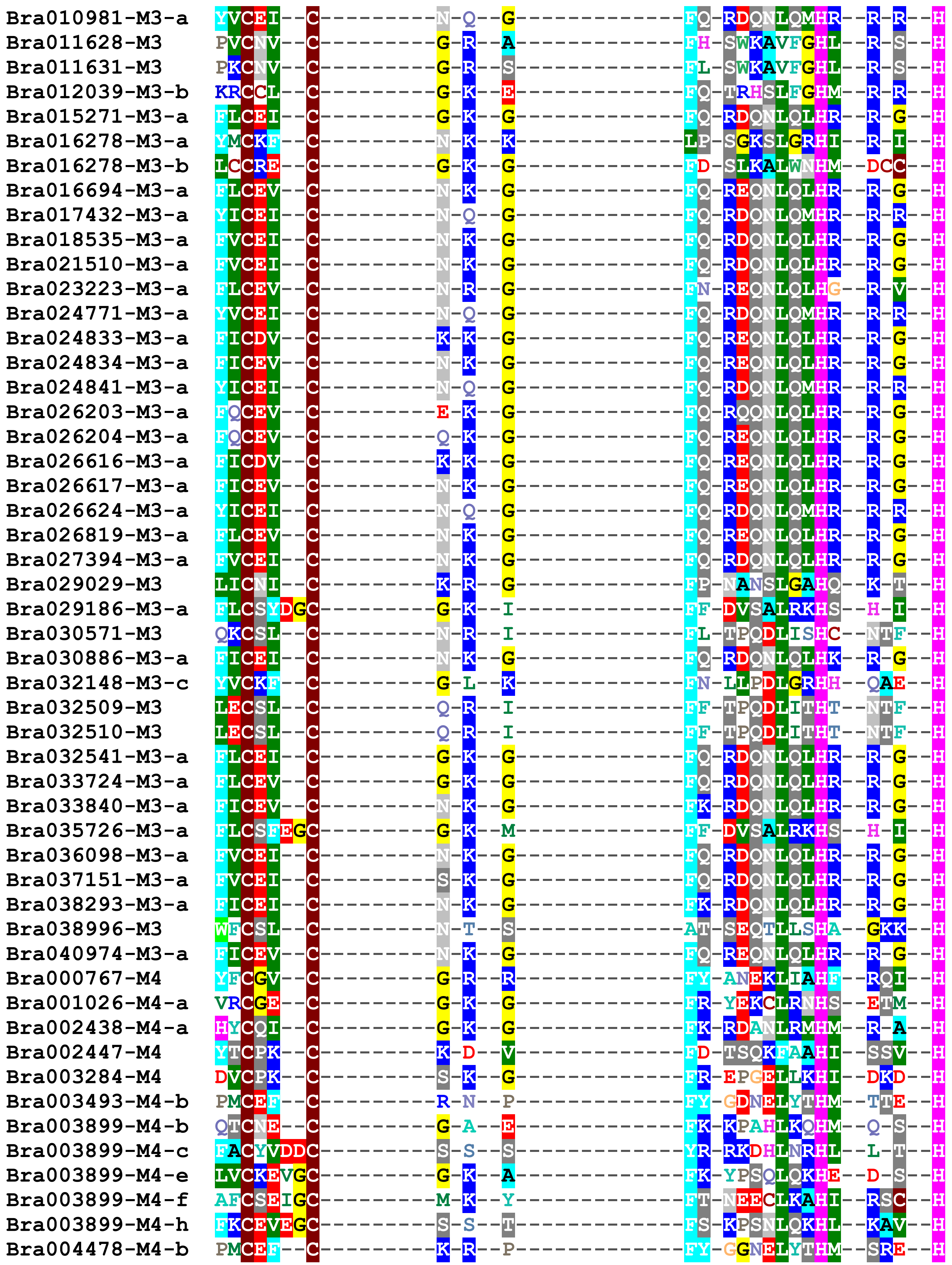

Bra010981-M3-a YVCEI--C---------N-Q--G-------------FQ-RDQNLQMHR--R-R--H
Bra011628-M3 PVCNV--C---------G-R--A-------------FH-SWKAVFGHL--R-S--H
Bra011631-M3 PKCNV--C---------G-R--S-------------FL-SWKAVFGHL--R-S--H
Bra012039-M3-b KRCCL--C---------G-K--E-------------FQ-TRHSLFGHM--R-R--H
Bra015271-M3-a FLCEI--C---------G-K--G-------------FQ-RDQNLQLHR--R-G--H
Bra016278-M3-a YMCKF--C---------N-K--K-------------LP-SGKSIGRHI--R-I--H
Bra016278-M3-b LCCRE--C---------G-K--G-------------FD-SLKALWNHM--DCC--H
Bra016694-M3-a FLCEV--C---------N-K--G-------------FQ-REQNLQLHR--R-G--H
Bra017432-M3-a YICEI--C---------N-Q--G-------------FQ-RDQNLQMHR--R-R--H
Bra018535-M3-a FVCEI--C---------N-K--G-------------FQ-RDQNLQLHR--R-G--H
Bra021510-M3-a FVCEI--C---------N-K--G-------------FQ-RDQNLQLHR--R-G--H
Bra023223-M3-a FLCEV--C---------N-R--G-------------FN-REQNLQLHG--R-V--H
Bra024771-M3-a YVCEI--C---------N-Q--G-------------FQ-RDQNLQMHR--R-R--H
Bra024833-M3-a FICDV--C---------K-K--G-------------FQ-REQNLQLHR--R-G--H
Bra024834-M3-a FICEV--C---------N-K--G-------------FQ-REQNLQLHR--R-G--H
Bra024841-M3-a YICEI--C---------N-Q--G-------------FQ-RDQNLQMHR--R-R--H
Bra026203-M3-a FQCEV--C---------E-K--G-------------FQ-RQQNLQLHR--R-G--H
Bra026204-M3-a FQCEV--C---------Q-K--G-------------FQ-REQNLQLHR--R-G--H
Bra026616-M3-a FICDV--C---------K-K--G-------------FQ-REQNLQLHR--R-G--H
Bra026617-M3-a FICEV--C---------N-K--G-------------FQ-REQNLQLHR--R-G--H
Bra026624-M3-a YICEI--C---------N-Q--G-------------FQ-RDQNLQMHR--R-R--H
Bra026819-M3-a FLCEV--C---------N-K--G-------------FQ-REQNLQLHR--R-G--H
Bra027394-M3-a FVCEI--C---------N-K--G-------------FQ-RDQNLQLHR--R-G--H
Bra029029-M3 LICNI--C---------K-R--G-------------FP-NANSIGAHQ--K-T--H
Bra029186-M3-a FLCSYDGC---------G-K--I-------------FF-DVSALRKHS--H-I--H
Bra030571-M3 QKCSL--C---------N-R--I-------------FL-TPQDLISHC--NTF--H
Bra030886-M3-a FICEI--C---------N-K--G-------------FQ-RDQNLQLHK--R-G--H
Bra032148-M3-c YVCKF--C---------G-L--K-------------FN-LLPDIGRHH--QAE--H
Bra032509-M3 LECSL--C---------Q-R--I-------------FF-TPQDLITHT--NTF--H
Bra032510-M3 LECSL--C---------Q-R--I-------------FF-TPQDLITHT--NTF--H
Bra032541-M3-a FLCEI--C---------G-K--G-------------FQ-RDQNLQLHR--R-G--H
Bra033724-M3-a FLCEV--C---------G-K--G-------------FQ-RDQNLQLHR--R-G--H
Bra033840-M3-a FICEV--C---------N-K--G-------------FK-RDQNLQLHR--R-G--H
Bra035726-M3-a FLCSFEGC---------G-K--M-------------FF-DVSALRKHS--H-I--H
Bra036098-M3-a FVCEI--C---------N-K--G-------------FQ-RDQNLQLHR--R-G--H
Bra037151-M3-a FVCEI--C---------S-K--G-------------FQ-RDQNLQLHR--R-G--H
Bra038293-M3-a FICEI--C---------N-K--G-------------FK-RDQNLQLHR--R-G--H
Bra038996-M3 WFCSL--C---------N-T--S-------------AT-SEQTLLSHA--GKK--H
Bra040974-M3-a FICEV--C---------N-K--G-------------FQ-REQNLQLHR--R-G--H
Bra000767-M4 YFCGV--C---------G-R--R-------------FY-ANEKLIAHF--RQI--H
Bra001026-M4-a VRCGE--C---------G-K--G-------------FR-YEKCLRNHS--ETM--H
Bra002438-M4-a HYCQI--C---------G-K--G-------------FK-RDANLRMHM--R-A--H
Bra002447-M4 YTCPK--C---------K-D--V-------------FD-TSQKFAAHI--SSV--H
Bra003284-M4 DVCPK--C---------S-K--G-------------FR-EPGELLKHI--DKD--H
Bra003493-M4-b PMCEF--C---------R-N--P-------------FY-GDNELYTHM--TTE--H
Bra003899-M4-b QTCNE--C---------G-A--E-------------FK-KPAHLKQHM--Q-S--H
Bra003899-M4-c FACYVDDC---------S-S--S-------------YR-RKDHLNRHL--L-T--H
Bra003899-M4-e LVCKEVGC---------G-K--A-------------FK-YPSQLQKHE--D-S--H
Bra003899-M4-f AFCSEIGC---------M-K--Y-------------FT-NEECLKAHI--RSC--H
Bra003899-M4-h FKCEVEGC---------S-S--T-------------FS-KPSNLQKHL--KAV--H
Bra004478-M4-b PMCEF--C---------K-R--P-------------FY-GGNELYTHM--SRE--H

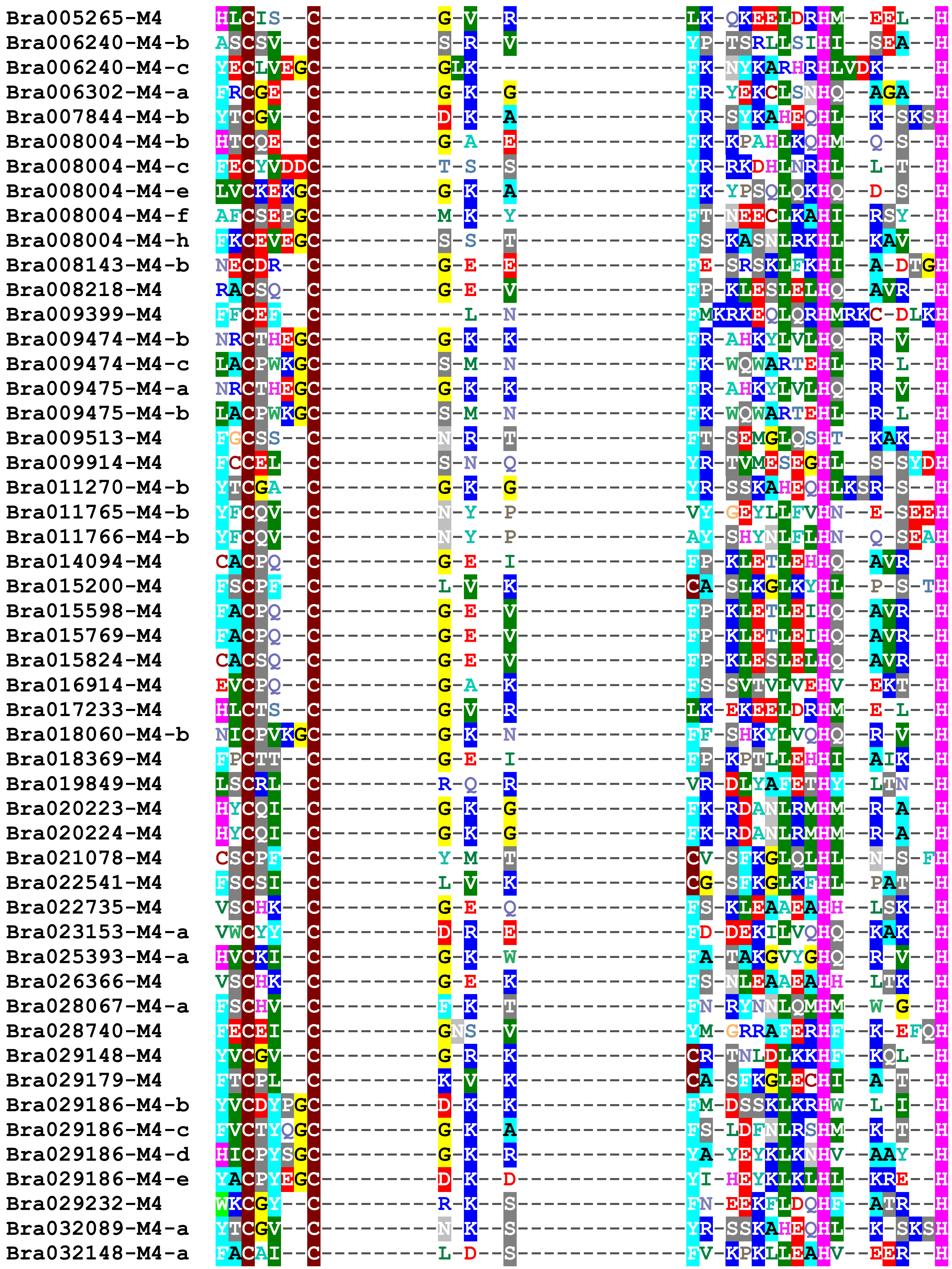
Bra005265-M4
Bra006240-M4-b
Bra006240-M4-c
Bra006302-M4-a
Bra007844-M4-b
Bra008004-M4-b
Bra008004-M4-c
Bra008004-M4-e
Bra008004-M4-f
Bra008004-M4-h
Bra008143-M4-b
Bra008218-M4
Bra009399-M4
Bra009474-M4-b
Bra009474-M4-c
Bra009475-M4-a
Bra009475-M4-b
Bra009513-M4
Bra009914-M4
Bra011270-M4-b
Bra011765-M4-b
Bra011766-M4-b
Bra014094-M4
Bra015200-M4
Bra015598-M4
Bra015769-M4
Bra015824-M4
Bra016914-M4
Bra017233-M4
Bra018060-M4-b
Bra018369-M4
Bra019849-M4
Bra020223-M4
Bra020224-M4
Bra021078-M4
Bra022541-M4
Bra022735-M4
Bra023153-M4-a
Bra025393-M4-a
Bra026366-M4
Bra028067-M4-a
Bra028740-M4
Bra029148-M4
Bra029179-M4
Bra029186-M4-b
Bra029186-M4-c
Bra029186-M4-d
Bra029186-M4-e
Bra029232-M4
Bra032089-M4-a
Bra032148-M4-a

```
Bra032148-M4-b    LQCIP--C---------G-S--N-------------FG-DKEQLLVHV--QAV--H
Bra033033-M4-a    NKCAA--C---------Y-K--Q-------------FN-KMEHLVEHM--KIS-YH
Bra034765-M4      FVCLF--C---------D-S--R-------------FV-SSDSLFDHC--RLS--H
Bra035726-M4-b    YVCDYPGC---------D-K--K-------------FL-DSSKLKRHW--L-I--H
Bra035726-M4-c    FVCTYEGC---------G-K--A-------------FS-LDFNLRSHM--K-T--H
Bra035726-M4-d    HICPYSGC---------G-K--R-------------YA-HEYKLKNHV--AAY--H
Bra035726-M4-e    YACPYEGC---------E-K--A-------------YI-HEYKLKLHL--KRE--H
Bra035938-M4-a    VLCTV--C---------Q-I--S-------------CI-SKEAYASHKYGK-R--H
Bra035938-M4-b    VWCQV--C---------Q-T--R-------------CN-SKAAYENHTYGK-K--H
Bra036591-M4      FYCEL--C---------S-K--Q-------------YR-TVMEFEGHL--D-SYDH
Bra036750-M4-a    HFCTI--C---------G-K--G-------------FK-RDANLRMHM--R-G--H
Bra037784-M4      FFCEF--C-----------L--N-------------FMKRKEQLQRHMRKC-DLKH
Bra039606-M4      YTCPK--C---------K-G--V-------------FN-TSQKFAAHM--S-S--H
Bra039880-M4      VWCYY--C---------D-R--E-------------FE-DEKILVQHQ--KAK--H
Bra039993-M4      FSCTT--C---------G-E--I-------------FP-KTNLLEHHF--AVK--H
Bra040971-M4-a    NFCTI--C---------G-K--G-------------FK-RDANLRMHM--R-G--H
Bra001416-M5      FNCSL--C---------H-F--G-------------TK-SIDQFRKHL--STDKEH
Bra023223-M5-c    WKCDK--C---------P-K--S-------------YA-LQSDWKTHSKICGIKVH
Bra003295-M5-a    FSCPV--C---------F-K--T-------------FN-RYNNMQMHM--W-G--H
Bra003810-M5      LYCIV--C---------S-K--N-------------FK-TEKQWKNHE--Q-SKKH
Bra003899-M5-a    YLCQY--C---------G-I--S-------------RS-KKYLITSHI--E-S--H
Bra003899-M5-d    FKCPVENC---------K-S--E-------------FS-IHGNISKHL--KKF--H
Bra003899-M5-i    FVCGFPGC---------G-M--R-------------FA-YKHVRNNHE--N-SGSH
Bra004050-M5-c    WICER--C---------S-K--G-------------YA-VQSDYKAHLKTCGTRGH
Bra004171-M5      RQCSS--C---------G-V--R-------------FK-CQEEHSKHM--D-W--H
Bra004283-M5-d    HSCD---C---------G-R--V-------------FS-RVESFIEHQ----D--H
Bra004324-M5      FHCEY--C---------S-L--D-------------CQ-SLNCFKEHLSTK-K--H
Bra004411-M5-a    FVCRA--C---------K-F--T-------------GL-SVASFTSHL--S-TEEH
Bra004411-M5-b    FLCGS--C---------N-F--T-------------AL-SVTDFSRHL--S-SEEH
Bra004414-M5-b    PRCGV--C---------F-K--H-------------CK-SFESVREHL--N-VPDH
Bra005232-M5      FYCLH--C---------D-R--Y-------------FC-NVSVRDDHF--K-TKKH
Bra005262-M5      TSCPF--C---------R-M--K-------------CG-SLKGVKHHL--T-S-SH
Bra005443-M5      WKCRI--C---------P-Q--V-------------NY-CFTDCKWHI--LDK--H
Bra005605-M5      YECKL--C---------L-T--L-------------HN-NEGNYLAHTQGK-R--H
Bra005752-M5      WLCRS--C---------S-Q--K-------------FS-TSEECTSHL--EQV--H
Bra006057-M5      HICWV--C---------L-D--F-------------FT-HQEPCYEHL--RAV--H
Bra006240-M5-a    ISCPIVGC---------P-E--R-------------LK-SLDNFEDHY--KAR--H
Bra006357-M5      YECPH--C-----------K--I-------------TAPDLKTMQIHH--ESK--H
Bra007346-M5      FSCPV--C---------F-K--T-------------FN-RYNNMQMHM--W-G--H
Bra007844-M5-a    LACNS--C---------N-K--E-------------FE-DDAEQKLHY--K-SEWH
Bra007844-M5-c    TCCLM--C---------D-K--K-------------HK-TLEKCMVHM--HKF--H
Bra008004-M5-a    YLCQY--C---------G-I--S-------------RS-KKYLITSHI--N-S--H
Bra008004-M5-d    FKCPVENC---------K-S--E-------------FS-VHGNISRHV--KKF--H
Bra008004-M5-i    FVCGFSGC---------A-K--R-------------FA-YKHVRNNHE--K-SGSH
Bra008143-M5-a    LYCIV--C---------S-K--K-------------FK-SEKQWKNHE--Q-SKKH
Bra008621-M5      YECPH--C-----------K--I-------------TAPDLKTMQIHH--ESK--H
Bra009038-M5      HICWV--C---------L-R--W-------------FA-QQEPCYEHL--RAV--H
Bra009474-M5-d    YKCKVEEC---------G-L--S-------------FR-FVSDYSRHR--R-KTGH
Bra009475-M5-c    YKCKVEEC---------G-L--S-------------FR-FVSDYSRHR--R-KTGH
Bra009629-M5      FICAAPHC---------L-R--S-------------FL-KKPDFESHV--HHL--H
Bra009785-M5-a    LCCKS--C---------N-F--D-------------CQ-SPEKFRKHL--S-SYKH
```

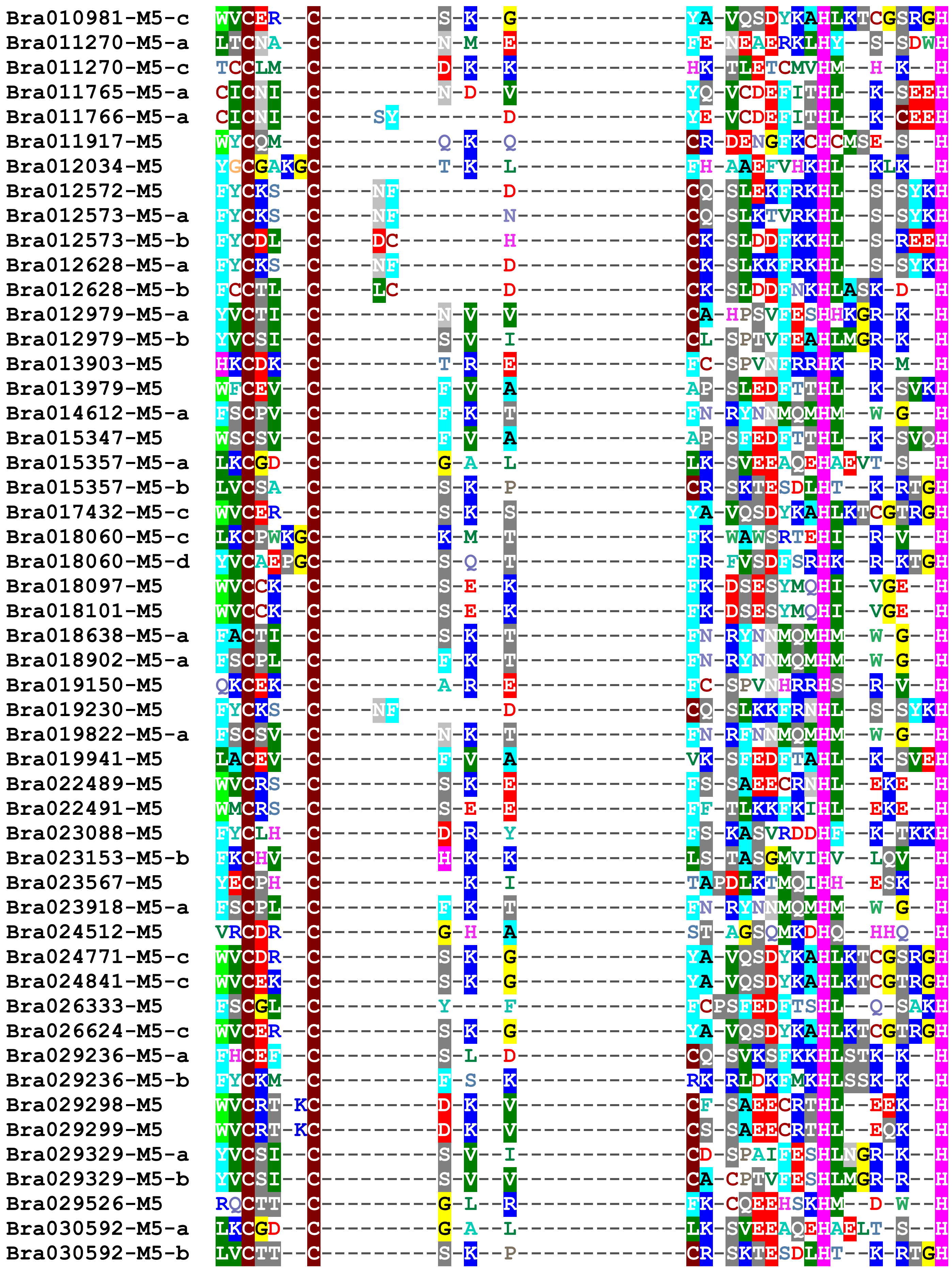
Bra010981-M5-c
Bra011270-M5-a
Bra011270-M5-c
Bra011765-M5-a
Bra011766-M5-a
Bra011917-M5
Bra012034-M5
Bra012572-M5
Bra012573-M5-a
Bra012573-M5-b
Bra012628-M5-a
Bra012628-M5-b
Bra012979-M5-a
Bra012979-M5-b
Bra013903-M5
Bra013979-M5
Bra014612-M5-a
Bra015347-M5
Bra015357-M5-a
Bra015357-M5-b
Bra017432-M5-c
Bra018060-M5-c
Bra018060-M5-d
Bra018097-M5
Bra018101-M5
Bra018638-M5-a
Bra018902-M5-a
Bra019150-M5
Bra019230-M5
Bra019822-M5-a
Bra019941-M5
Bra022489-M5
Bra022491-M5
Bra023088-M5
Bra023153-M5-b
Bra023567-M5
Bra023918-M5-a
Bra024512-M5
Bra024771-M5-c
Bra024841-M5-c
Bra026333-M5
Bra026624-M5-c
Bra029236-M5-a
Bra029236-M5-b
Bra029298-M5
Bra029299-M5
Bra029329-M5-a
Bra029329-M5-b
Bra029526-M5
Bra030592-M5-a
Bra030592-M5-b

```
Bra030450-M5-a   FSCPL--C---------F-K--T-------------FN-RYNNMQMHM--W-G--H
Bra030860-M5     WYCQM--C---------Q-K--Q-------------CR-DENGFKCHCMSE-S--H
Bra031239-M5-a   FSCPL--C---------Y-K--T-------------FN-RYNNMQMHM--W-G--H
Bra031621-M5-a   FACSI--C---------S-K--T-------------FT-RYNNMQMHM--W-G--H
Bra032089-M5-b   TSCLM--C---------D-K--K-------------HK-TIEKCMVHM--HKL--H
Bra032130-M5-a   FWCDL--C---------S-V--G-------------AC-NETVMRNHELGK-K--H
Bra032130-M5-b   IWCEA--C----NI--------V-------------TY-SENVMETHKLGK-K--H
Bra033384-M5     QTCSV--C---------D-D--G-------------GM-SYWLHNRHV--T-I--H
Bra034322-M5     YGCGAKGC---------T-K--L-------------FH-AAEFVHKHL--KLK--H
Bra034366-M5     YVCEV--C---------G-N--K-------------VL-RGRHEWDHH--TQGRVH
Bra036435-M5     HFCGL--C---------D-L--S-------------FP-SFDDFTTHF--K-TPPH
Bra038687-M5     WSCSV--C----HY--------D-------------AP-TFEDFTKHL--K-CETH
Bra039412-M5     YFCRV--C---------D-C--V-------------VK-DSANYLDHINGK-K--H
Bra039880-M5     FKCHA--C---------H-K--K-------------LS-SASGMVIHV--LQV--H
Bra040199-M5     WMCRT--C---------A-Q--K-------------FF-YVKKFRNHI--EQE--H
Bra040200-M5     WLCRT--C---------S-R--K-------------FF-YVKKFRNHL--EQE--H
Bra040607-M5     PVCRV--C---------N-V--V-------------LK-SESLWDVHQASR-K--H
Bra001523-Z1-b   YVCPEASC----VHHDPT-R--A-------------LG-DLTGIKKHF--CRK--H
Bra002438-Z1-b   YSCPHQGC-------RWN----Q------RHEKFQPL-KSVICAKNHY--KRS--H
Bra002438-Z1-c   YMCTR--C---------NVK--H-------------FS-VLSDLRTHE--K----H
Bra003140-Z1     FKCEI--C---------GNQ--S-------------YK-GRRSFKMHF--K-EAQH
Bra003493-Z1-a   LSCSV--C----------DK--AEEEQGPGPRHRLRIK-SVEQLKGHL--YHK--H
Bra003493-Z1-c   YTCHI--C----QRSQTG-KYEY-------------YK-NYDDLELHF--RRD--H
Bra003493-Z1-d   FLCEDDSC--------LSKKFIV-------------FP-NESELKRHN--AIE--H
Bra004050-Z1-b   YVCPEPTC----LHHNPC-H--A-------------LG-DLVGIKKHF--RRK--H
Bra004283-Z1-b   YVCPEPTC----LHHNPC-H--A-------------LG-DLVGIKKHF--RRK--H
Bra004478-Z1-a   LSCTS--C------VSNHRM--R-------------FK-SVEHLKNHL--NHQ--H
Bra004478-Z1-c   YTCHI--C----QRLRPG-QYEY-------------YG-NYDDLEAHF--RSD--H
Bra004478-Z1-d   FLCEDESC--------LAKKFIV-------------FQ-TEPELKRHN--TVD--H
Bra005741-Z1-b   YICPIKTC----VHHDSS-R--A-------------LG-DLTGIKKHY--SRK--H
Bra005882-Z1     FKCEI--C---------RNK--I-------------YK-GRRAFERHF--K-ESQH
Bra007844-Z1-d   FICLY--C---------NELRHP-------------FS-SLEAVRKHMEAK-S--H
Bra009549-Z1-b   YICPIKTC----VHHDSS-R--A-------------LG-DLTGIKKHY--SRK--H
Bra009785-Z1-b   LYCKV--CCGGQGP--------D-------------YK-SLDNLRTHL--S-SEEH
Bra010981-Z1-b   YVCPEPTC----LHHDPC-H--A-------------LG-DLVGIKKHF--RRK--H
Bra011270-Z1-d   FMCLY--C---------SELCHA-------------FS-SLEAVRKHMEAK-S--H
Bra016694-Z1-b   YLCPEPTC----VHHDPS-R--A-------------LG-DLTGIKKHY--YRK--H
Bra017432-Z1-b   YVCPEPTC----LHHAPC-H--A-------------LG-DLVGIKKHF--RRK--H
Bra018638-Z1-b   YCCAE-GC----KNNINHPRAKP-------------LK-DFRTLQTHY--KRK--H
Bra019822-Z1-b   YCCAE-GC-KNNIDHPRA-K--P-------------LK-DFRTLQTHY--KRK--H
Bra021510-Z1-b   YVCPEASC----VHHDPS-R--A-------------LG-DLTGIKKHF--CRK--H
Bra023223-Z1-b   FICPEPTC----VHHDPS-H--A-------------LR-DFTGIKKHY--YRK--H
Bra024771-Z1-b   YVCPEPTC----LHHNPC-H--A-------------LG-DLVGIKKHF--RRK--H
Bra024833-Z1-b   YICPEPTC----VHHDPA-R--A-------------LG-DLTGVKKHY--YRK--H
Bra024834-Z1-b   YLCPEPSC----VHHDPS-R--A-------------LG-DLTGIKKHY--YRK--H
Bra024841-Z1-b   YVCPEPSC----LHHDPC-H--A-------------LG-DLVGIKKHF--RRK--H
Bra025591-Z1-a   YHCSI--C---------DFSIDL-------------YC-ARNPLELVV-HYPK-GH
Bra025591-Z1-b   YSCMH--C----DDFVLH-E--T-------------CA-NLPRKQRHVLHN----H
Bra026203-Z1-b   YVCPEPTC----VYHDPS-R--A-------------LG-DLTGIKKHY--CRK--H
Bra026204-Z1-b   YVCPEPTC----VYHDPS-R--A-------------LG-DLTGIKKHY--YRK--H
Bra026616-Z1-b   YLCPEPTC----VHHDHS-R--A-------------LG-DLTGIKKHY--YRK--H
```

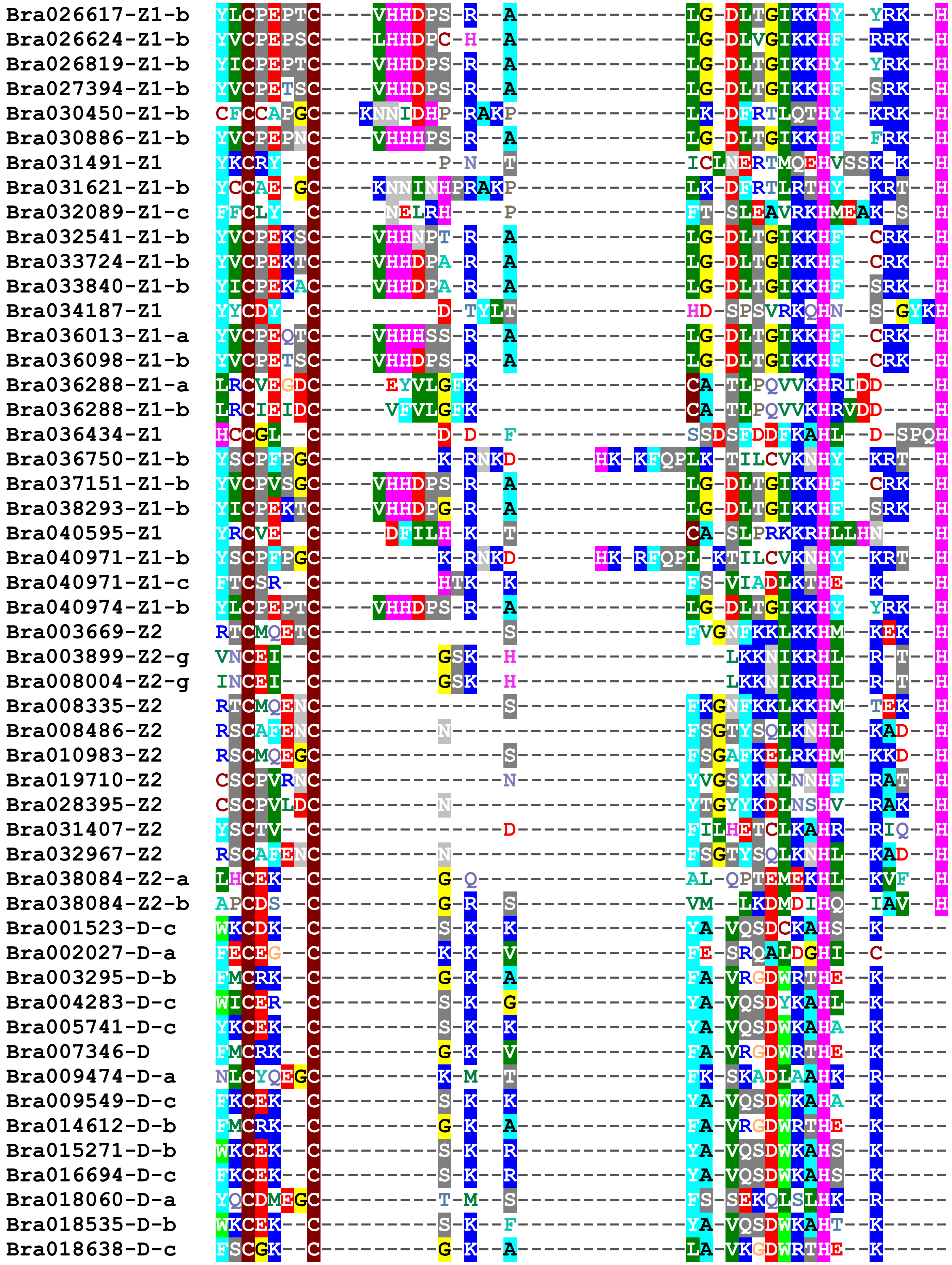
Bra026617-Z1-b
Bra026624-Z1-b
Bra026819-Z1-b
Bra027394-Z1-b
Bra030450-Z1-b
Bra030886-Z1-b
Bra031491-Z1
Bra031621-Z1-b
Bra032089-Z1-c
Bra032541-Z1-b
Bra033724-Z1-b
Bra033840-Z1-b
Bra034187-Z1
Bra036013-Z1-a
Bra036098-Z1-b
Bra036288-Z1-a
Bra036288-Z1-b
Bra036434-Z1
Bra036750-Z1-b
Bra037151-Z1-b
Bra038293-Z1-b
Bra040595-Z1
Bra040971-Z1-b
Bra040971-Z1-c
Bra040974-Z1-b
Bra003669-Z2
Bra003899-Z2-g
Bra008004-Z2-g
Bra008335-Z2
Bra008486-Z2
Bra010983-Z2
Bra019710-Z2
Bra028395-Z2
Bra031407-Z2
Bra032967-Z2
Bra038084-Z2-a
Bra038084-Z2-b
Bra001523-D-c
Bra002027-D-a
Bra003295-D-b
Bra004283-D-c
Bra005741-D-c
Bra007346-D
Bra009474-D-a
Bra009549-D-c
Bra014612-D-b
Bra015271-D-b
Bra016694-D-c
Bra018060-D-a
Bra018535-D-b
Bra018638-D-c

```
Bra018902-D-b   FACRR--C---------G-K--A-------------FA-VKGDWRTHE--K-----
Bra021510-D-c   WKCDK--C---------S-K--K-------------YA-VQSDCKAHS--K-----
Bra023918-D-b   FACRR--C---------G-K--A-------------FA-VKGDWRTHE--K-----
Bra024833-D-c   WKCDK--C---------S-K--R-------------YA-VQSDWKAHS--K-----
Bra024834-D-c   WKCEK--C---------S-K--R-------------YA-VQSDWKAHS--K-----
Bra026203-D-c   FKCEK--C---------D-K--F-------------YA-VESDCKAHS--K-----
Bra026204-D-c   FKCEK--C---------N-K--F-------------YA-VESDCKAHF--K-----
Bra026616-D-c   WKCEK--C---------S-K--R-------------YA-VQSDWKAHS--K-----
Bra026617-D-c   WKCEK--C---------S-K--R-------------YA-VQSDWKAHS--K-----
Bra026819-D-c   WNCEK--C---------S-K--R-------------YA-VQSDWKAHS--K-----
Bra027394-D-c   WKCDK--C---------S-K--K-------------YA-VQSDCKAHS--K-----
Bra028067-D-b   YACRI--C---------G-K--L-------------LA-VKGDWRTHE--K-----
Bra030450-D-c   FACRR--C---------G-K--A-------------FA-VKGDWRTHE--K-----
Bra030886-D-c   WKCDK--C---------S-K--K-------------YA-VQSDWKAHA--K-----
Bra031239-D-b   FACRR--C---------G-K--A-------------FA-VKGDWRTHE--K-----
Bra031621-D-c   FSCGK--C---------G-K--A-------------LA-VKGDWRTHE--K-----
Bra031820-D     FVCEI--C---------N-K--G-------------FQ-RDQNLRLHR--R-----
Bra032541-D-c   WKCGK--C---------S-K--R-------------YA-VQSDWKAHS--K-----
Bra033033-D-b   PTCGV--C---------K-K--H-------------CR-SFESLREHL--I-----
Bra033724-D-c   WTCEK--C---------A-K--R-------------YA-VHSDYKAHS--K-----
Bra033840-D-c   WKCDK--C---------S-K--K-------------YA-VMSDWKAHS--K-----
Bra036013-D-b   WKCEK--C---------A-K--R-------------YA-VLSDYKAHS--K-----
Bra036098-D-c   WKCDK--C---------S-K--R-------------YA-VVSDWKAHS--K-----
Bra037151-D-c   FKCEK--C---------S-K--S-------------YA-VQSDWKAHS--K-----
Bra038293-D-c   WKCDK--C---------S-K--K-------------YA-VVSDWKAHS--K-----
Bra040600-D     FECPH--C-----------K--I-------------TAPDLKTMQIHHESR-----
Bra040974-D-c   WKCEK--C---------S-K--R-------------YA-VQSDWKAHS--K-----
```
